# The 18 kDa Translocator Protein, Microglia and Neuroinflammation

**DOI:** 10.1111/bpa.12196

**Published:** 2014-10-26

**Authors:** Guo‐Jun Liu, Ryan J. Middleton, Claire R. Hatty, Winnie Wai‐Ying Kam, Ronald Chan, Tien Pham, Meredith Harrison‐Brown, Eoin Dodson, Kelly Veale, Richard B. Banati

**Affiliations:** ^1^ Life Sciences Australian Nuclear Science and Technology Organisation NSW Australia; ^2^ Brain & Mind Research Institute The University of Sydney NSW Australia; ^3^ Discipline of Medical Imaging & Radiation Sciences Faculty of Health Sciences The University of Sydney NSW Australia; ^4^ National Imaging Facility and Ramaciotti Brain Imaging Centre Sydney NSW Australia

**Keywords:** microglia, neuroinflammation, PBR111, PET, PK11195, TSPO

## Abstract

The 18 kDa translocator protein (TSPO), previously known as the peripheral benzodiazepine receptor, is expressed in the injured brain. It has become known as an imaging marker of “neuroinflammation” indicating active disease, and is best interpreted as a nondiagnostic biomarker and disease staging tool that refers to histopathology rather than disease etiology. The therapeutic potential of TSPO as a drug target is mostly based on the understanding that it is an outer mitochondrial membrane protein required for the translocation of cholesterol, which thus regulates the rate of steroid synthesis. This pivotal role together with the evolutionary conservation of TSPO has underpinned the belief that any loss or mutation of TSPO should be associated with significant physiological deficits or be outright incompatible with life. However, against prediction, full *Tspo* knockout mice are viable and across their lifespan do not show the phenotype expected if cholesterol transport and steroid synthesis were significantly impaired. Thus, the “translocation” function of TSPO remains to be better substantiated. Here, we discuss the literature before and after the introduction of the new nomenclature for TSPO and review some of the newer findings. In light of the controversy surrounding the function of TSPO, we emphasize the continued importance of identifying compounds with confirmed selectivity and suggest that TSPO expression is analyzed within specific disease contexts rather than merely equated with the reified concept of “neuroinflammation.”

## Microglia and Brain Imaging

**Figure 1:**
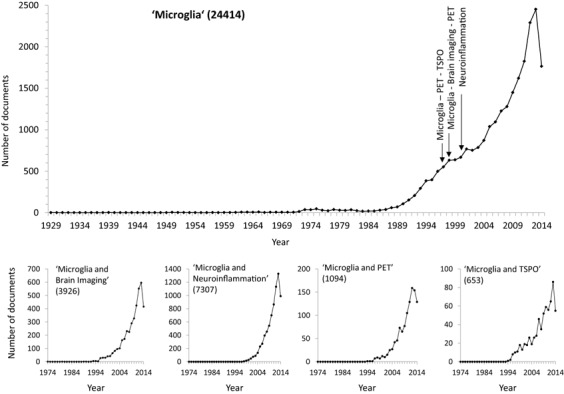
Figure 1 shows the increase in microglia literature. There is a steep rise from around 1995 in the overall publication activity under the heading of “microglia” that coincides with emergence of the themes “microglia and neuroinflammation” (7307 publications), “microglia and brain imaging” (3926 publications), “microglia and PET” (1094 publications) and “microglia and TSPO” (653 publications). The arrows in the upper, larger panel indicate the appearance of the first articles (1997–2000) retrieved under the specified search terms.

**Figure 2:**
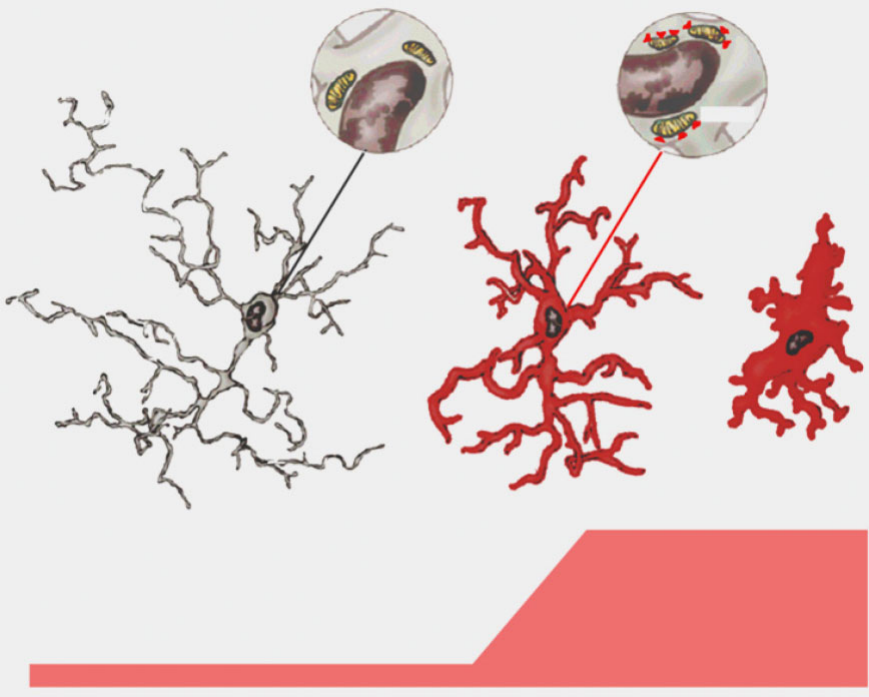
Adapted from 16. The transition of parenchymal microglia from their normal state to an activated state is accompanied by the *de novo* expression of binding sites for (R)‐PK11195, the prototypical ligand originally used to describe the presence and function of TSPO 100. Transformation of microglia into ameboid cells, such as seen after injuries involving neuronal cell death 20, does not appear to lead to any further increase in TSPO expression as measured by binding of (R)‐PK11195. This implies that the dynamic range of signals based on the expression of TSPO is best exploited at the point of transition from resting to activated, but not ameboid, microglia.

**Figure 3:**
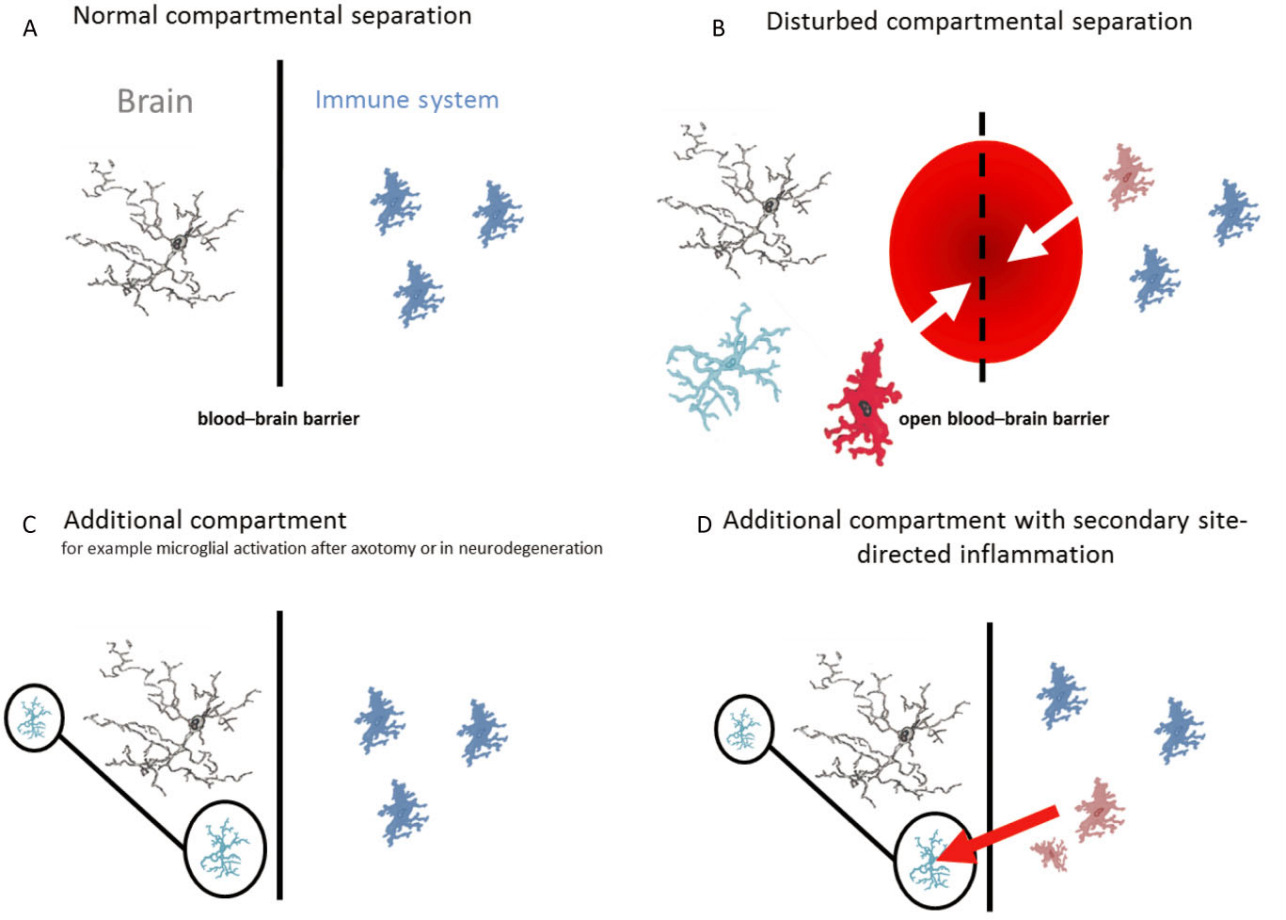
Adapted from 17. (**A**) The blood–brain barrier (BBB) separates the brain from the peripheral immune system. TSPO expression in the brain is low to absent. (**B**) Pathologies causing disruption of the BBB allow the influx of cells of the peripheral immune system. TSPO expression may be due to activated microglia or invading blood‐borne macrophages. (**C**) Noninflammatory neuronal injuries without obvious BBB damage, such as after peripheral nerve lesion, evoke microglial responses locally and in projection areas. TSPO expression occurs throughout a network of neural tracts. (**D**) The presence of activated microglia can confer regional “immune alertness” with the secondary site‐directed recruitment of, for example, circulating T‐lymphocytes. The neuronally triggered glial response may evolve toward a delayed inflammatory response.

**Figure 4:**
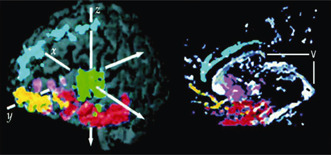
From 37. The 3D [11C](R)‐PK11195 binding potential map projected onto the magnetic resonance image (MRI) of a patient with herpes encephalitis indicates how the inflammation in the temporal lobe and hippocampus (red) is associated with TSPO expression that tracks throughout the limbic system beyond the focal injury. This is likely to be due to Wallerian degeneration along the affected neural tract system (blue = anterior cingulate; green = thalamus and brainstem; purple = insular cortex; yellow = orbitofrontal gyri; the MRI subtraction image in the right panel shows the volume losses, including the enlargement of the ventricles (v) of the destructive tissue pathology).

Active brain disease causes a change in the functional state of microglia. This state change is associated with the *de novo* expression of the mitochondrial 18 kDa translocator protein (TSPO; or peripheral benzodiazepine receptor; PBR), a binding site for which selective high‐affinity compounds were developed in the early 1980s 27, 99, 100, 101. This discovery was the starting point for the systematic development of *in vivo* imaging of cellular (nonneuronal) brain pathology (Figure 1). The principles underlying brain imaging based on detecting binding sites, such as TSPO, that are predominantly expressed by microglia focally and along anatomical neural pathways have received much attention in recent years [15, 16, 20; Figures 2, 3 and 4].

**Figure 5:**
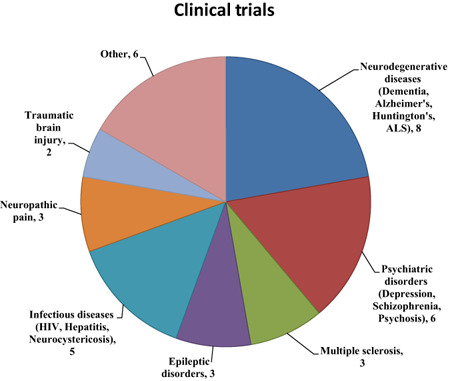
Clinical trials listed under “neuroinflammation” on clinicaltrials.gov as of August 1, 2014.

The sustained interest in TSPO ligands for neuroimaging is due to the fact that during active disease, the expression of this protein is upregulated from a very low baseline. This is in direct contrast to neuroreceptors which are usually downregulated, their loss often correlating with neurological or cognitive deficits. However, increased regional TSPO expression in the brain typically covaries with disease state and activity rather than clinical deficit and, therefore, does not map easily onto established disease classifications. In this sense, TSPO is a nondiagnostic biomarker that is best interpreted in the context of histopathology and only secondary to disease etiology. A substantial number of clinical trials are now being conducted, the majority of which are using *in vivo* measurements of TSPO expression as biomarkers of disease progression or therapeutic efficacy (Figure 5; Table S1 in Supporting Information).

The lexical change (Table 1) from the operational definition of “peripheral benzodiazepine receptor or binding site” to “18 kDa translocator protein TSPO” emphasizes one physiological function, the “translocation” of cholesterol and essential rate determining step in the synthesis of steroids, over other proposed functions. Research into the therapeutic potential of TSPO generally assumes this to be the operant mechanism of drug action 23, 151, 152, 153.

**Table 1:** Aliases for translocator protein (18 kDa)

Abbreviation	Name
PBR	Peripheral benzodiazepine receptor
PTBR	Peripheral‐type benzodiazepine receptor
MBR	Mitochondrial benzodiazepine receptor
IBP	Isoquinoline binding protein
PBBS	Peripheral benzodiazepine binding site
BPBS	Benzodiazepine peripheral binding site
Bzrp	Benzodiazepine receptor (peripheral)
Mdrc	Mitochondrial DBI receptor complex
DBI	Diazepam binding inhibitor
pk18	18 kDa PK (isoquinoline) binding subunit
Pkbs	PK (or isoquinoline) binding site
ω 3	Omega 3 site
TSPO	Translocator protein (18 kDa)

This review, however, is written at a time when new data have become available which show that knocking out the *Tspo* gene does not result in the phenotype expected if steroid synthesis was indeed altered 19, 123, 163, 170. While there are clear limitations, even with regard to global *Tspo* knockouts, to how far it is possible to extrapolate to the protein's function in the context of a normal organism, it suggests that the “translocation” concept needs to be rethought. Like the “neuroinflammation” concept, the concept of “translocation” is critically dependent on the context within which it is applied.

Here, we provide an overview of the publication trends before and after the introduction of the new nomenclature for TSPO, the efforts to develop new TSPO ligands as well as some of the newer findings. This review emphasizes that the continued development of new or the reassessment of existing molecules, with confirmed selectivity for TSPO, will be crucial to the further development of the field at large.

## The 18 kDa TSPO, Formerly Known as PBR

The 18 kDa TSPO has been implicated in many important physiological functions, including cholesterol transport and steroidogenesis 94, 125, 134, cellular respiration 77 and immunomodulation 102. Increased binding of ligands selective for TSPO has been observed at sites of central nervous system (CNS) pathology, such as glioma 162, 165, 166, 179, multiple sclerosis 21 as well as Alzheimer's disease (AD) 38, and TSPO ligand binding is now seen as a hallmark for microglial activation *in vivo*. These observations have led to a surge in the development of TSPO‐specific ligands appropriate for imaging and with potential for clinical applications. So far, none of these are in routine use in the clinic, but the number of clinical trials recently initiated (Table S1 in Supporting Information) indicates this may change in the years to come.

Although much has been learned about this protein's molecular, biochemical and pharmacological properties, its exact role under physiological and pathological conditions rests on a number of assumptions which have come to be doubted in the wake of the recent discovery that mice with tissue‐specific or global knockout of *Tspo* are, counter to expectation and prior indication in the literature, neither embryonic lethal nor obviously impaired in their production of steroids 123, 159, 170. The phenotype of these animals appears to contradict a significant proportion of the so far accepted evidence. As a note of caution, however, it is, important to realize that constitutive or conditional gene knockout allows for compensatory mechanisms either during early development or through the presence of alternative pathways, and thus do not necessarily falsify all claims about the role of TSPO in the adult organism. In any case, 2014 marks a potentially pivotal year in TSPO research and exciting controversial debate is imminent.

Given these recent developments in the field and the abundance of reviews on the protein, it is not our intention to discuss all of these proposed functions in detail; the reader is directed to other articles 59, 67, 164 for a comprehensive overview. In this review, we present a selection of the relevant literature, some prior to the name change in 2006 136 and some prior to the general acceptance of cholesterol translocation as TSPO's defining function, and summarize the efforts in developing selective TSPO‐binding compounds for use in neuroimaging.

## Historical Aspects

Literature relating to TSPO encompasses many research fields, highlighting a diverse range of reported functions and pathological involvement, and the inherent complexity this entails. A timeline of the early observations about TSPO prior to 2006 [before the name change; 136] can be seen in Table 2.

**Table 2:** A selection of early discoveries in the TSPO field.Abbreviations: ACTH = adrenocorticotropic hormone; AD = Alzheimer's disease; ADC = adenine nucleotide carrier; ANT = adenine nucleotide translocator; CSF = cerebrospinal fluid; DRG = dorsal root ganglion; MPTP = mitochondrial permeability transition pore; PBR = peripheral benzodiazepine receptor; PET = positron emission tomography; TNF = tumor necrosis factor; TSPO = 18 kDa translocator protein; VDAC = voltage‐dependent anion channel

Year	Discovery	Author/s
1977	Existence of peripheral benzodiazepine binding site	Braestrup & Squires 34
1981	Availability of radiolabeled Ro5‐4864, binding in rat cerebral cortex	Schoemaker *et al* 154
1982	Demonstrates distinct population of Ro5‐4864 binding in brain, predominantly on nuclear fraction	Marangos *et al* 112
1982	Localization of PBR to glia using Ro5‐4864	Schoemaker *et al* 156
1983	First use of PK11195 *in vitro*	Le Fur *et al* 99
1983	First use of PK11195 *in vivo*	Le Fur *et al* 100
1983	Shows Ro5‐4864 is an agonist, PK11195 an antagonist	Le Fur *et al* 101
1983	Autoradiography demonstrates presence in olfactory bulb, median eminence, choroid plexus and ependyma (CSF)	Benavides *et al* 27
1983	Ro5‐4864 binding in kidney and cerebral cortex (nuclear fraction) of rat	Schoemaker *et al* 155
1985	Hypophysectomy reduces Ro5‐4864 binding in testis and adrenal gland, expression localised to cell type reliant on pituitary input	Anholt *et al* 3
1985	Distribution in neonate rats similar to adults, associated with mitochondria and oxidative phosphorylation	Anholt *et al* 4
1985	Differential and discrete localisation of PBR in endocrine organs	De Souza *et al* 48
1986	TSPO localizes to outer membrane of mitochondria	Anholt *et al* 5
1987	Porphyrins are endogenous ligands of TSPO	Verma *et al* 174
1989	PBR possibly involved in adrenal ACTH stimulated pregnenolone formation	Besman *et al* 29
1990	PBR functionally linked to Leydig cell steroidogenesis, possibly acting on cholesterol	Papadopoulos *et al* 138
1990	PBR linked to rate limiting step (cholesterol translocation) of steroidogenesis	Krueger & Papadopoulos 94
1992	18 kDa PBR protein associates with VDAC and ADC (ANT)	McEnery 119
1993	PBR may modulate inflammatory response in brain through effects on cytokine release	Taupin *et al* 167
1996	PBR ligands induce cell death via MPTP in presence of TNF	Pastorino *et al* 141
1998	Cholesterol recognition amino acid consensus sequence of PBR identified	Li & Papadopoulos 103
1999	Arsenite induces apoptosis via MPTP, an effect potentiated by PK11195	Larochette *et al* 98
2000	PK11195 used in PET imaging of brain pathology	Banati *et al* 21
2001	PK11195 as PET tool for imaging microglial activation in early stages of AD	Cagnin *et al* 37
2002	PBR gene expression increased in DRG in peripheral axotomy model of neuropathic pain	Xiao *et al* 180

TSPO was discovered in 1977 by virtue of its ability to bind the benzodiazepine diazepam in peripheral tissues 34. At this time, the benzodiazepine Ro5‐4864 was also identified as a ligand for peripheral sites based on its inability to block binding of diazepam at the central benzodiazepine receptor (CBR), while blocking binding to an additional site in mitochondria of peripheral tissues 34. Schoemaker *et al* 154 were among the first to use this ligand for the characterization of the protein. The subsequent discovery of 1‐(2‐chlorophenyl)‐N‐methyl‐(1‐methylpropyl)‐3 isoquinoline carboxamide (PK11195), a nonbenzodiazepine, TSPO‐selective ligand, by PHARMUKA Laboratories (Gennevilliers, France) 100 enabled a significant increase in research productivity in characterizing the protein. In the early 1990s, TSPO research began to increase in popularity, coinciding with the publication of the first data implicating TSPO in cholesterol transport and steroid synthesis 29, 94, 125, 138. The protein was also cloned around this time 140, 161. Another notable rise in the rate of publications can be observed around the year 2000 when [3H]PK11195 binding was definitively localized to activated microglia in the CNS 21, leading to the recognition of PK11195 binding as a hallmark of microglial activation *in vivo* and a subsequent increase in its use as an imaging tool in the clinical neurosciences (“*in vivo* neuropathology”). Peaks in publication in subsequent years also reflect an increased interest in TSPO ligands as therapeutic tools in various pathological conditions, including anxiety 153 and cancer 110.

Initially named the “peripheral” or “peripheral‐type benzodiazepine receptor” as well as the number of other terms (Table 1), the protein was renamed in 2006 by the “working group for renaming the PBR” supported by Novartis (Basel, Switzerland). It proposed “translocator protein (18 kDa)” or TSPO, together with prefixes, mitoTSPO or nucTSPO, to denote either mitochondrial or nuclear location, as the new nomenclature 136. The new nomenclature was argued for on several grounds: (i) the transport, or “translocation” of cholesterol as the preeminent role of the protein over alternative functions, notably in energy metabolism 77, 133, 182; (ii) the sequence homology with the tryptophan‐rich sensory protein family (TspO) that function as light, oxygen and stress‐sensitive regulators of photosynthesis genes; and (iii) the applicability of the name “TSPO” across species, noting that the rat gene had been shown to substitute for its *Rhodobacter sphaeroides* homolog TspO 182.

While this name change created clarity by restricting to a single identifier, it marked a significant shift away from operational definitions, such as PK11195 binding site or peripheral‐type benzodiazepine binding site, that were more cautious 66 in assigning a “functional” definition to the protein. This new name signalled the rationale for the first clinical phase II trials into the efficacy, safety and tolerability of Emanupil (XBD173 Novartis; AC‐5216 Dainippon Pharmaceutical), a new compound believed to modulate the “translocation”, and thus steroid regulating function of TSPO. A trial in patients with generalized anxiety disorder was registered in 2005 (http://clinicaltrials.gov/show/NCT00108836) 91, 152.

## Key Trends in the TSPO Literature

**Figure 6:**
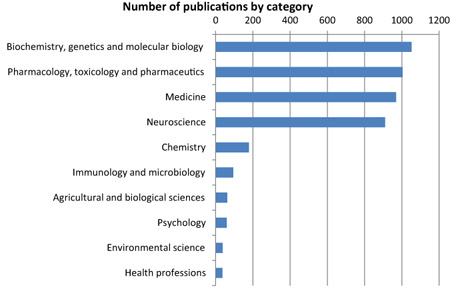
Top 10 SCOPUS research areas by citation rates of TSPO‐related articles. Neuroscience is one of the leading areas for TSPO research.

**Figure 7:**
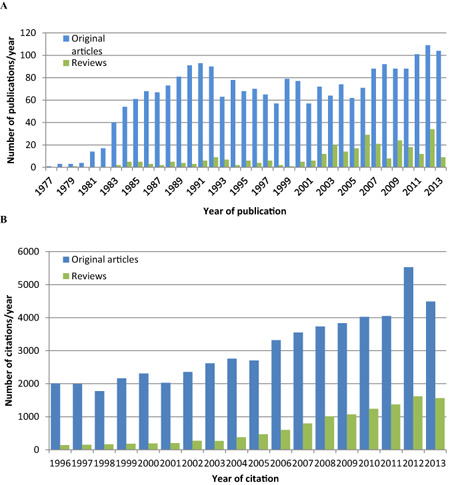
(**A**) Publication of articles relating to TSPO per year demonstrates two waves of publication, roughly corresponding to protein/binding site characterization and later application of ligands for imaging and preclinical studies. (**B**) Citation of articles relating to TSPO have increased in the wake of potential applications of ligands.

The precise examination of the relevant literature is challenging because of the sheer number of publications as well as the ambiguity and diversity in names for the protein. Here, we have used SCOPUS, one of the largest available online databases of peer‐reviewed scientific literature, to reveal trends in publishing and citation rates of articles and the most prominent subject areas in which TSPO‐related research occurs. These bibliometric data are indicative only as there are inherent limitations of automated databases, including inconsistencies between the available services 1, 13, 57, 105. A search of the SCOPUS database returned a total of 2763 journal articles relating to TSPO (the individual search terms and corresponding publication and citation rates are listed in Table S2 and Figure S1 in Supporting Information). Of these, approximately 89% were original articles and 11% were reviews. The vast majority of research relating to TSPO falls into one or more of four categories: biochemistry, genetics and molecular biology; pharmacology, toxicology and pharmaceutics; medicine; and neuroscience (Figure 6). These broad categories may be roughly translated to research into the cellular functions of the protein, applications of targeted drugs, research into the role of the protein in disease, and more specifically, the role of the protein in diseases of the nervous system. This demonstrates that much of the interest in TSPO comes from researchers in the field of neuroscience and mental health. However, it should be noted that despite the change in nomenclature, a number of groups still use other names either in addition to or instead of the term “TSPO”. Therefore, literature searches intended to capture the entire field, including foundational literature before the introduction of the new nomenclature, require the use of several search terms. The overall publications trends are shown in Figure 7 and the most cited publications are listed in Table 3.

**Table 3:** Top 20 original articles ranked in order of citation rates. Citation information accessed through SCOPUS database

Author	Year	Title	Journal	Times cited
Cagnin A., Brooks D.J., Kennedy A.M., Gunn R.N., Myers R., Turkheimer F.E., Jones T., Banati R.B.	2001	*In‐vivo* measurement of activated microglia in dementia	Lancet	503
Mcenery M.W., Snowman A.M., Trifiletti R.R., Snyder S.H.	1992	Isolation of the mitochondrial benzodiazepine receptor: Association with the voltage‐dependent anion channel and the adenine nucleotide carrier	Proceedings of the National Academy of Sciences of the United States of America	453
Taupin V., Toulmond S., Serrano A., Benavides J., Zavala F.	1993	Increase in IL‐6, IL‐1 and TNF levels in rat brain following traumatic lesion. Influence of pre‐ and post‐traumatic treatment with Ro5 4864, a peripheral‐type (p site) benzodiazepine ligand	Journal of Neuroimmunology	340
Anholt R.R.H., Pedersen P.L., De Souza E.B., Snyder S.H.	1986	The peripheral‐type benzodiazepine receptor. Localization to the mitochondrial outer membrane	Journal of Biological Chemistry	330
Banati R.B., Newcombe J., Gunn R.N., Cagnin A., Turkheimer F., Heppner F., Price G., Wegner F., Giovannoni G., Miller D.H., Perkin G.D., Smith T., Hewson A.K., Bydder G., Kreutzberg G.W., Jones T., Cuzner M.L., Myers R.	2000	The peripheral benzodiazepine binding site in the brain in multiple sclerosis. Quantitative *in vivo* imaging of microglia as a measure of disease activity	Brain	325
Gerhard A., Pavese N., Hotton G., Turkheimer F., Es M., Hammers A., Eggert K., Oertel W., Banati R.B., Brooks D.J.	2006	*In vivo* imaging of microglial activation with [11C](R)‐PK11195 PET in idiopathic Parkinson's disease	Neurobiology of Disease	270
Ouchi Y., Yoshikawa E., Sekine Y., Futatsubashi M., Kanno T., Ogusu T., Torizuka T.	2005	Microglial activation and dopamine terminal loss in early Parkinson's disease	Annals of Neurology	243
Li H., Papadopoulos V.	1998	Peripheral‐type benzodiazepine receptor function in cholesterol transport. Identification of a putative cholesterol recognition/interaction amino acid sequence and consensus pattern	Endocrinology	224
Hirsch T., Decaudin D., Susin S.A., Marchetti P., Larochette N., Resche‐Rigon M., Kroemer G.	1998	PK11195, a ligand of the mitochondrial benzodiazepine receptor, facilitates the induction of apoptosis and reverses Bcl‐2‐mediated cytoprotection	Experimental Cell Research	220
Turner M.R., Cagnin A., Turkheimer F.E., Miller C.C.J., Shaw C.E., Brooks D.J., Leigh P.N., Banati R.B.	2004	Evidence of widespread cerebral microglial activation in amyotrophic lateral sclerosis: An [11C](R)‐PK11195 positron emission tomography study	Neurobiology of Disease	209
Cicchetti F., Brownell A.L., Williams K., Chen Y.I., Livni E., Isacson O.	2002	Neuroinflammation of the nigrostriatal pathway during progressive 6‐OHDA dopamine degeneration in rats monitored by immunohistochemistry and PET imaging	European Journal of Neuroscience	209
Hardwick M., Fertikh D., Culty M., Li H., Vidic B., Papadopoulos V.	1999	Peripheral‐type benzodiazepine receptor (PBR) in human breast cancer: Correlation of breast cancer cell aggressive phenotype with PBR expression, nuclear localization, and PBR‐mediated cell proliferation and nuclear transport of cholesterol	Cancer Research	205
Krueger K.E., Papadopoulos V.	1990	Peripheral‐type benzodiazepine receptors mediate translocation of cholesterol from outer to inner mitochondrial membranes in adrenocortical cells	Journal of Biological Chemistry	186
Li H., Yao Z.‐X., Degenhardt B., Teper G., Papadopoulos V.	2001	Cholesterol binding at the cholesterol recognition/interaction amino acid consensus (CRAC) of the peripheral‐type benzodiazepine receptor and inhibition of steroidogenesis by an HIV TAT‐CRAC peptide	Proceedings of the National Academy of Sciences of the United States of America	175
Yiangou Y., Facer P., Durrenberger P., Chessell I.P., Naylor A., Bountra C., Banati R.R., Anand P.	2006	COX‐2, CB2 and P2X7‐immunoreactivities are increased in activated microglial cells/macrophages of multiple sclerosis and amyotrophic lateral sclerosis spinal cord	BMC Neurology	172
Papadopoulos V., Mukhin A.G., Costa E., Krueger K.E.	1990	The peripheral‐type benzodiazepine receptor is functionally linked to Leydig cell steroidogenesis	Journal of Biological Chemistry	163
Banati R.B., Myers R., Kreutzberg G.W.	1997	PK (“peripheral benzodiazepine”)—binding sites in the CNS indicate early and discrete brain lesions: microautoradiographic detection of [3H]PK 11195 binding to activated microglia	Journal of Neurocytology	159
Vowinckel E., Reutens D., Becher B., Verge G., Evans A., Owens T., Antel J.P.	1997	PK11195 binding to the peripheral benzodiazepine receptor as a marker of microglia activation in multiple sclerosis and experimental autoimmune encephalomyelitis	Journal of Neuroscience Research	157
Carayon P., Portier M., Dussossoy D., Bord A., Petitprêtre G., Canat X., Le Fur G., Casellas P.	1996	Involvement of peripheral benzodiazepine receptors in the protection of hematopoietic cells against oxygen radical damage	Blood	153
Stephenson D.T., Schober D.A., Smalstig E.B., Mincy R.E., Gehlert D.R., Clemens J.A.	1995	Peripheral benzodiazepine receptors are colocalized with activated microglia following transient global forebrain ischemia in the rat	Journal of Neuroscience	151

## The *Tspo* Gene

The human *Tspo* gene is located on chromosome 22q13.3. It consists of four exons and encodes 169 amino acids 106, 150. An alternatively spliced variant named *PBR‐S* lacks exon 2 and contains an open reading frame that differs to TSPO 106. Despite the observation that *PBR‐S* mRNA is about 10 times more abundant than *Tspo*, there is currently no experimental evidence for the existence of a PBR‐S protein. Recent studies have found that a single‐nucleotide polymorphism in exon 4, which causes a nonconservative alanine to threonine substitution in the TSPO protein, has an effect on ligand‐binding affinity 93, 122, 132. Thus, this genetic polymorphism must be considered when performing quantitative imaging of TSPO using positron emission tomography (PET).

Characterization of the *Tspo* promoter has revealed that it resides within a CpG island and contains GC boxes but lacks TATA or CCAAT elements 24, 106. A detailed description of *Tspo* transcriptional regulation can be found in a recent review 25. In addition to promoter‐based transcriptional control, a short interspersed repetitive element of the B2 family has been found to generate an antisense transcript that downregulates expression of *Tspo* in mouse cells 59.

Despite being evolutionarily conserved from bacteria to humans, there are organisms such as *Escherichia coli* and the yeast *Saccharomyces cerevisiae* that do not contain *Tspo* in their genomes 103, 149. Fan *et al* have recently provided a review of the likely evolution of TSPO 58. Ginter *et al* 69 suggest, based on the observations in bacterial TspO, that the original role of the protein may relate to porphyrin catabolism and the consumption of reactive oxygen species (ROS), while cholesterol transport (translocation) is a “moonlight function” found in higher organisms.

## Structure of the TSPO Protein

An understanding of the structure of the TSPO protein provides valuable insight into its function and interaction with ligands, a knowledge that is essential in the development of pharmacological interventions that target TSPO. The current model of TSPO describes it as an integral membrane protein consisting of five transmembrane alpha helices, an extramitochondrial C‐terminal, an intramitochondrial N‐terminal, two extramitochondrial loops and two intramitochondrial loops, for which Jaremko *et al* have recently been able to provide confirmatory data 84.

### Structure of the TSPO monomer

The five putative transmembrane domains of TSPO were initially indicated by hydropathy analysis of the human TSPO amino acid sequence, which indicated five hydrophobic stretches that were each predicted to span one layer of the membrane bilayer 28. The use of site‐directed mutagenesis and labeling techniques provided further evidence of five transmembrane domains; however, it was predicted that these regions would span the entire membrane bilayer 85. Early molecular dynamics simulations predicted that the five transmembrane regions had an alpha helix structure 28. This has been supported by a nuclear magnetic resonance (NMR) study of peptide fragments representing the five putative transmembrane regions of the mouse TSPO 126. Furthermore, circular dichroism spectroscopy analysis of the whole mouse TSPO protein revealed an alpha helical content of 45%, which is close to the value of 53% predicted if transmembrane domains make up 100 of 169 amino acids in the TSPO sequence 126. It is also interesting to note that the binding of the TSPO ligand PK11195 increases the helical content from 45% to 52% 126. While the tertiary fold of the whole TSPO in detergent micelles was too unstable for further NMR study of tertiary structure, the addition of a high concentration of PK11195 did have a stabilizing effect 126.

TSPO has a short intramitochondrial N‐terminal and a longer extramitochondrial C‐terminal 28, 85. Currently, there is a lack of information on the structure of the N‐terminal. However, considerable effort has gone into modeling the C‐terminal because of its potential role as a TSPO‐binding site for cholesterol 82, 103, 104. A cholesterol recognition/interaction amino acid consensus sequence (CRAC) has been identified on the C‐terminal of TSPO, supporting its involvement in cholesterol transport 103. NMR analysis of a TSPO C‐terminal peptide (amino acids 144–169) indicated a helical conformation for the L144 to S159 fragment of the peptide and a groove that could accommodate a cholesterol molecule 82. Analysis of the amino acid sequence also indicates that the C‐terminal is highly charged 28, 85.

The five transmembrane domains of TSPO are joined by two extramitochondrial and two intramitochondrial loops. Differential labeling of amino acids indicated that loops one and three were located on the cytoplasmic side of the membrane, while loops two and four were facing the inside of the mitochondria 85, in agreement with earlier predictions 28. However, structural information about these loops is currently less detailed than for the transmembrane domains and experimental studies of the structure of the loop regions are still lacking. Investigations into the binding sites of TSPO ligands have provided some additional information about the interhelix loops and are described in more detail in the succeeding text.

The functional model first proposed by Bernassau *et al* 28 supports the hypothesis that TSPO can act as a cholesterol transporter. It also indicates that TSPO has a channel structure to facilitate this transport. Molecular dynamics simulations indicated that the five transmembrane alpha helices may form a channel approximately 3–4 Å wide, which could accommodate a cholesterol molecule 28. Homology modeling using the crystal structure of apolipophorin III, and docking with cholesterol using shape fitting, further indicates that TSPO monomer forms a molecular channel that can transport a single cholesterol molecule 152. Furthermore, the 10‐Å resolution structure of bacterial homologue TspO provides evidence that the transmembrane alpha helices are arranged to form a channel structure for the translocation of molecules across the membrane 92. Recent successful structure determination using the prototypical TSPO ligand PK11195 to stabilize the protein 84 substantially validates earlier predictions of the structure of TSPO.

### Monomeric and oligomeric arrangement of TSPO

TSPO is functional as a monomer 95; however, there is also evidence that it forms oligomeric complexes with itself and other proteins 49, 168. TSPO monomers are able to bind PK11195 and cholesterol 95 and it has also been suggested that TSPO forms homo‐oligomers which may be important in cholesterol binding and transport 49, 168. The formation of oligomers has been correlated with an increased presence of ROS, which are thought to induce covalent bonding of TSPO monomers 49. In addition, recent transmission electron microscope images of negatively stained mouse TSPO reconstituted into lipid have revealed the presence of circular objects that are likely to be the association of at least four TSPO monomers 168.

There is evidence that the bacterial homologue TspO from *R. sphaeroides* forms homodimers in the presence of protoporphyrinogen 181, which is further supported by structural evidence that TspO forms dimers when reconstituted into helical crystals 92. In both mammals and bacteria, it has been proposed that the formation of TSPO oligomers is a dynamic process that may be associated with ligand binding and transport function 49, 181; however, some function can still be retained in its monomeric form 49, 95.

The stabilizing effect of PK11195 on the protein reported by Jaremko *et al* 84 was not seen with bacterial TspO, suggesting that the PK11195 interaction relates to the cholesterol‐associated functions of the protein expressed in mammals, and is due to differences in the tertiary and quaternary structure of TSPO not seen in bacteria 83.

### TSPO association with other proteins

While TSPO can function as an isolated monomer or in homo‐oligomers, in mitochondria it is also believed to form a trimeric complex with the 32 kDa voltage‐dependent anion channel (VDAC) and the 30 kDa adenine nucleotide carrier 119. TSPO has also been implicated as part of the mitochondrial permeability transition pore (MPTP), reflecting its putative role in apoptosis 172. Early studies also revealed a possible complex with a 10 kDa 30 and 43 kDa 47 protein that was labeled by high‐affinity ligands for the 18 kDa TSPO; however, the identity of these proteins has not been elucidated. Additionally, there is evidence that TSPO interacts with other proteins and peptides including peripheral benzodiazepine receptor‐associated protein 1 (PRAX‐1) 65, steroidogenic acute regulatory protein (StAR) 76, peripheral benodiazepine receptor‐associated protein (PAP7) 108 and diazepam binding inhibitor (DBI) 71 (Table S3 in Supporting Information).

### Ligand binding sites

In addition to binding putative endogenous ligands such as cholesterol and protoporphyrin IX, TSPO binds a range of synthetic ligands. These compounds encompass a range of structural classes including benzodiazepines, isoquinoline carboxamide derivatives, imidazopyridines, benzothiazepines, benzoxazepines, indole acetamide derivatives, phenoxyphenylacetamide derivatives, pyrazolopyrimidines and vinca alkaloids 81.

**Figure 8:**
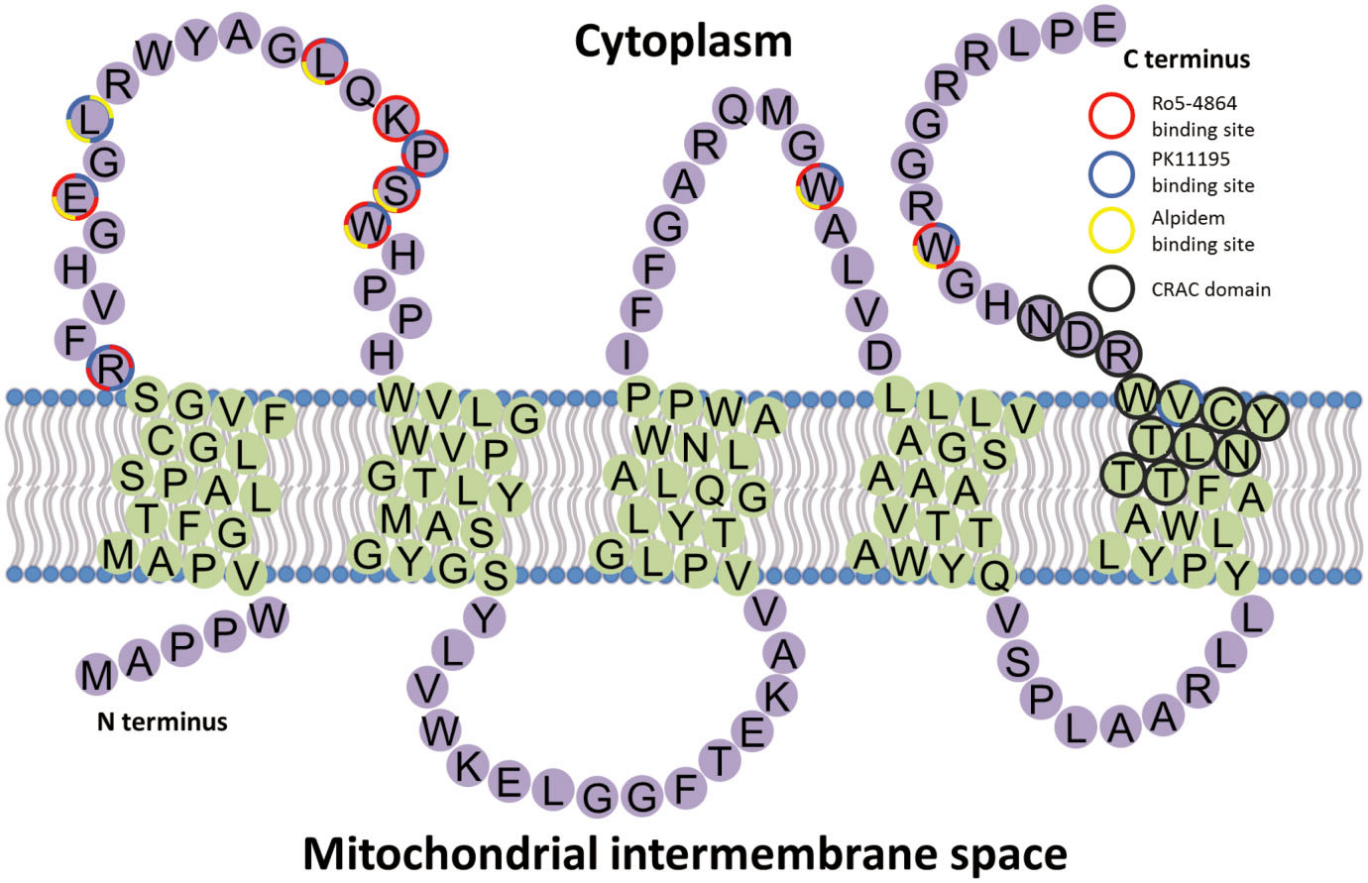
The topology of TSPO in the membrane, with amino acids involved in the binding of Ro5‐4863, PK11195, Alpidem and the CRAC domain highlighted.

The binding sites of the benzodiazepine Ro5‐4864 and the isoquinoline carboxamide derivative PK11195 on TSPO have been investigated using site‐directed mutagenesis, which revealed that the two ligands have distinct but overlapping binding sites that involve amino acid residues on the first cytoplasmic loop and the C‐terminal 60 (Figure 8). Further predictions of ligand binding sites on TSPO were made using molecular modeling and analysis of structure–activity relationships 9. The binding site for Ro5‐4864 likely includes the residues R24, G29, L37, K39, P40, S41, W42, W107 and W161, while the PK11195 binding site includes the residues R24, G29, L31, L37, P40, S41, W42, W107 and W161 9. Furthermore, the binding site of the imidazopyridine alpidem is predicted to include the residues G29, L31, L37, S41, W42, W107 and W161 9. While molecular modeling has provided a useful prediction of the structure of the ligand binding sites, experimental studies of the structure of the loop regions are still lacking.

## Functional Roles of TSPO

### Cholesterol transport and steroid biosynthesis

Although TSPO has been linked to many physiological processes, including immunomodulation 102, metabolism and cellular respiration 5, 77, by far the most widely accepted role of this protein is the one in cholesterol transport and steroid synthesis. Since the first observations were made 29, 94, 125, 138, this idea has been extensively explored.

The fact that TSPO is located in steroid‐synthesizing cell types in endocrine organs itself suggested a role in endocrine function. Although the steroidogenic process had been well characterized previously, there were still lingering questions. While at the time it was known that steroid synthesis takes place in mitochondria and requires cleavage of cholesterol into pregnenolone by the cytochrome P‐450 side‐chain cleavage enzyme on the inner mitochondrial membrane, it was still unclear how cholesterol is transported from the outer to inner mitochondrial membranes, a process found to be the rate‐limiting step for this reaction 147. Being located on the outer mitochondrial membrane (OMM) of steroid‐synthesizing cells, TSPO became a potential candidate for fulfilling this role.

An important first study that explored this was that by Papadopoulos *et al* 138, which measured the production of progesterone in MA‐10 Leydig cells in the presence of known TSPO ligands including the isoquinoline carboxamides PK11195, PK14067, PK14068, as well as the benzodiazepines Ro5‐4864, diazepam, flunitrazepam, clonazepam, zolpidem and flumazenil at a range of concentrations. These ligands not only increased secretion of progesterone, but also did so to a degree that correlates with TSPOs pharmacological profile (i.e., PK11195 increased progesterone production the most, with flumazenil having no effect). A subsequent study using isolated mitochondria found that PK11195 stimulated hormone synthesis at the mitochondrial level by acting on cholesterol already available in the OMM, as addition of excess cholesterol to media did not enhance steroid synthesis 94. The same study employed digitonin titration of mitochondrial membranes to demonstrate that PK11195 exerted its effects by enhancing translocation of cholesterol from outer to inner mitochondrial membranes, the rate‐limiting step in steroid synthesis. Antisense knockout studies have also linked TSPO to steroid production 90. A cholesterol recognition site, identified by deletion of the cytosolic carboxyl terminal of TSPO, has been identified, and termed the CRAC domain 103. These studies brought about a surge in publication activity based on this new theory.

However, it is still unclear whether TSPO is necessary or sufficient for steroid synthesis. Multiple pathways for cholesterol transport have been suggested 120, with the protein StAR now being shown to play a crucial role 121. An essential steroidogenic role for StAR is clear, as mice with the gene knocked out die soon after birth of adrenocortical insufficiency 40, and many humans with congenital lipoid adrenal hyperplasia possess a nonfunctional StAR gene 107.

Although mutations in the *Tspo* gene have not been linked directly to disease states involving impaired steroid production, the *Tspo* gene is evolutionarily highly conserved 45, 181 which has been interpreted as indicating a nonredundant, integral function for the organism. While earlier reports of embryonic lethal outcomes from *Tspo* knockout studies indeed appeared to confirm this assumption 135, more recent work shows that *Tspo* is an ancient but nonessential gene 18, 19, 123, 159, 163, 170. In light of the evolutionary pressure to conserve the gene, its documented influence on steroidogenesis and the previously observed embryonic lethality caused by the knockout of either *Tspo* 135 or the endogenous PBR/TSPO ligand, Acyl‐CoA binding protein 97, the survival and overtly normal phenotype of a global *Tspo* knockout animal might seem unexpected. However, the evolutionary preservation of an ancient gene does not necessarily imply that it is essential, nor that an ancient and nonessential gene is less likely to be relevant for the emergence of disease. On the contrary, phylostratigraphic analysis of the human genome suggests that nonessential ancient genes predominate among the disease‐associated genes 53. *PBR*/*Tspo* may therefore be considered a nonessential disease gene that lies at the functional periphery of the interactome, with more limited direct interactions than an *in utero* essential hub gene 22. Consequently, the functional impact on a *Tspo* knockout would be predicted to be discreet.

It has been hypothesized that TSPO is involved in the production of steroids in the brain 137. Neurosteroids have been found to exert anxiolytic effects by allosterically modulating γ‐amino butyric acid (GABA) neurotransmission 151. Altered PK11195 binding has previously been found on platelets 176 and lymphocytes 61, 62 of patients with anxiety disorders. Together, these data suggest that TSPO plays a role in neurosteroid production, hence affecting anxiety states, making it a potential target for anxiolytic drugs. New TSPO ligands have been shown to have anxiolytic effects in animal models 46, 153 and human trials 153. High doses of the ligand XBD173 were shown to exert quick acting anxiolytic effects without sedation and withdrawal effects in a male cohort subjected to cholecystokinin tetrapeptide‐induced anxiety 153, while the ligand N,N‐di‐n‐propyl‐2‐(4‐methylphenyl)indol‐3‐ylglyoxylamide was shown to stimulate allopregnanolone production and exert anxiolytic effects in rats subjected to the elevated plus maze 46. However, XBD173 has been demonstrated by PET to bind with mixed affinity in the human brain 131, raising the possibility that its anxiolytic effects involve binding sites other than TSPO.

### TSPO in cell cycle and cellular bioenergetics

Early studies found that the binding site for isoquinoline carboxamides (such as PK11195 and irreversible photosensitive ligand PK14105) was an 18 kDa protein that formed complexes with a number of other proteins 51, 109, 118, 119. Two proteins of 30 kDa and 32 kDa were subsequently confirmed to be the VDAC and the adenine nucleotide carrier (also known as the adenine nucleotide translocator), two candidate regulatory components of the MPTP 119. The MPTP is a nonspecific pore that opens in response to mitochondrial stress and allows the free passage of molecules under 1.5 kDa, resulting in the uncoupling of oxidative phosphorylation 74. Thus, the MPTP is a crucial regulator of cell death, in particular by apoptosis 98, 141. A series of studies has demonstrated a modulating effect of TSPO ligands on apoptosis in cell culture [for reviews see 172, 173], which indicates that the protein may have a role in modulating the mitochondrial permeability transition. This implication of a role in apoptosis is significant, as increased TSPO expression has been demonstrated in neoplastic cell types and in tumors 162. The potential for TSPO ligands to induce apoptosis in neoplastic cells that have increased expression of the protein has made it a promising target for anti‐tumorigenic therapies. PK11195 has already been used experimentally as an anti‐tumor agent 2, 96. However, a recent study has called much of this evidence into doubt; the group found that mitochondria isolated from mouse livers with a conditional deletion of *Tspo* were just as capable of undergoing the permeability transition as the controls, suggesting TSPO may not have as integral a role as once thought (159).

It has recently been reported that TSPO also has a role in modulating gluconeogenesis, and thus has relevance for the pathophysiology of obesity and diabetes. While screening the effect of 2400 bioactive compounds on gluconeogenesis in zebrafish 73, the TSPO ligands PK11195 and Ro5‐4864 were identified as compounds that decrease glucose and reduce energy consumption, suggesting a role for TSPO in glucose metabolism. Gut *et al* 73 further demonstrated that PK11195 and Ro5‐4864 (in micromolar concentrations) reduced blood glucose in 24‐h fasted mice, and reduced weight gain and improved glucose tolerance in a mouse model of high‐fat diet‐induced obesity and diabetes 73. The effect of PK11195 on glucose homeostasis suggests that TSPO may have a role in cellular energy consumption, which may relate to the function of mitochondria in energy production (e.g., adenosine triphosphate (ATP) production). This could explain the seemingly conflicting evidence derived from steroidogenic cell lines, as changes in bioenergetics are likely to indirectly affect other cell functions including steroid synthesis. However, at this stage, the selectivity of the observed effects of the TSPO ligands PK11195 and Ro5‐4864 remain to be tested at lower (nanomolar) concentrations. Likewise, the selectivity and specificity of the effects of the ligands to TSPO require further investigation.

## TSPO Ligand Development

### Early characterization of selective ligands

**Figure 9:**
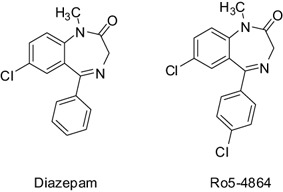
Structures of diazepam and Ro5‐4864.

TSPO was originally identified indirectly by the pharmacological profile of its binding sites: (i) clonazepam acting as a weak inhibitor (opposite to the central‐type binding site); (ii) diazepam's affinity was significantly lower; and (iii) Ro5‐4864 potently inhibited binding (Figure 9). It was not until the availability of [3H]Ro5‐4864 in 1981 that TSPO could be identified directly in experiments utilizing radioligand binding and autoradiography. The subsequent discovery of the nonbenzodiazepine ligand PK11195 and its ability to potently displace Ro5‐4864 from its binding sites 27, 99, 100, 101 increased the scope of exploration. Although initial thermodynamic studies indicated that Ro5‐4864 was most likely to be an agonist or partial agonist, and PK11195 an antagonist 101, subsequent studies have shown PK11195 to have similar actions to Ro5‐4864 138. With these two tools, it became possible to begin to derive the physiological and pharmacological significance of TSPO, especially because they were both found to bind *in vivo* 99.

TSPO has been found to have a ubiquitous organ distribution, including adrenal gland, kidney, heart, platelets 154, lung, testis, liver and muscle 112 and brown fat 4. In contrast to central benzodiazepine binding sites, expression of TSPO is generally low in the healthy brain, found mainly in the olfactory bulb, and nonparenchymal regions such as ependyma and choroid plexus 27, 68. A significant increase in binding can be seen under pathological conditions 156. Although the homogenization of tissue required for radioligand binding prevented the easy identification of the specific cell types responsible for binding in the organs mentioned earlier, autoradiography has proven to be a more helpful tool, demonstrating increased binding in areas including the cortex of the adrenal gland, ventricular tissue in the heart 4, interstitial tissue (including Leydig cells) in the testis 48 and the distal tubules of the cortex and ascending limb of the loop of Henle in the kidney 68. Hypophysectomy in rats showed that TSPO is generally expressed on cells in the adrenal gland and testis that rely on trophic influence from the pituitary gland and produce steroid hormones 3.

Evidence for the subcellular localization of TSPO is conflicting. An early study showed co‐distribution of TSPO ligand binding with cytochrome oxidase activity that alluded to mitochondrial involvement 4, with subsequent studies using subcellular fractionation techniques directly indicating the presence of TSPO ligand binding on the mitochondrial fraction 5, 6. Although generally accepted as a mitochondrial membrane protein, evidence suggests that there may be other subcellular locations for TSPO, including nucleus 112, 155, plasma membrane 128 and an undefined microsomal compartment 124, 127. Whether the difference in localization is due to technical differences or the tissue tested remains unclear. It is interesting to note that PK11195 and Ro5‐4864 both bind to erythrocytes, even though these cells have no mitochondria or nucleus 129. However, it is safe to say that TSPO has a primarily mitochondrial location and this has been an important factor in subsequent studies.

Ligands have again proven useful in determining the precise mitochondrial location of TSPO. Digitonin titration with mitochondrial isolates, a previously well‐documented technique, was used by Anholt *et al* 5 to demonstrate the presence of PK11195 binding on the OMM by its co‐release with the OMM marker enzyme, monoamine oxidase. Other studies have confirmed this using nondigitonin techniques 127. However, a study by Mukherjee and Das 124 used both digitonin titration and osmotic shock to isolate mitochondrial membrane compartments and found that TSPO binding was present on the inner mitochondrial membrane (IMM). High resolution electron microscopy images would be helpful for clarifying the true location(s) of TSPO binding.

The above‐mentioned ligands have been used to explore many facets of TSPO. A photosensitive isoquinoline carboxamide derivative, PK14105, was found to displace PK11195 and Ro5‐4864 and was used to isolate and visualize the 18 kDa TSPO protein by gel electrophoresis 7, 51. The mechanism by which PK14105 irreversibly cross‐links to the TSPO protein is unknown, but is assumed to be a nucleophilic aromatic substitution by an amino acid side chain functional group oriented in close proximity to the bound ligand 51. This ligand was later used to isolate two important proteins associated with TSPO 119. PK11195 and Ro5‐4864 have also been used to confirm cloning of rat 161 and bovine 140 TSPO and as a tool for cloning human *Tspo* 150. They have also been used to explore TSPO's physiological significance, including the protein's most well‐characterized role in steroid synthesis. These two ligands have experienced varying use over time. While PK11195 has been used extensively throughout the last 30 years, publications using Ro5‐4864 have been on the decline since the early 1990s. This can be largely attributed to the high affinity of PK11195 to TSPO across multiple species 12. Use of both ligands has declined recently with the onset of a new league of ligands that address drawbacks of the former ligands while maintaining high affinity. Nonetheless, both are still considered the prototype ligands to which all others are compared and much has been learned about TSPO through their past and continued use.

### Development of new synthetic ligands

With TSPO being implicated in so many physiological functions and pathologies, extensive resources have been dedicated to the discovery of new synthetic molecules with high affinity and selectivity for the protein. Although the original intention was to develop molecules as potential therapeutics by acting as modulators of TSPO pharmacology, the emergence of radio‐ and photoaffinity‐labeled analogues as part of TSPO characterization has resulted in exciting pharmacological, biochemical and *in vivo* imaging applications. With respect to the latter, a significant volume of literature relates to TSPO imaging applications in inflammation, such as multiple sclerosis and infection, as well as neurodegenerative disorders, cancer and heart disease.

The prototypical ligands for TSPO, Ro5‐4864 and PK11195, have been invaluable tools for the molecular and biochemical characterization of the TSPO protein. In addition to potential therapeutic applications, the carbon‐11 analogues [11C]Ro5‐4864 and [11C]PK11195 have been used to measure, quantify and image increased TSPO receptor overexpression in neurodegenerative disorders 26 and in neoplastic tissue 162. TSPO overexpression levels in platelets have also been examined as potential indicators of anxiety and stress 54, 176 while [125/131I]‐PK11195 have been explored as potential imaging/radiotherapeutic agents 2, 177.

However, initial PET imaging with [11C]Ro5‐4864 in patients with brain tumors did not show great promise because of large amounts of nonspecific binding and low *in vitro* affinity in human brain tissue 12. On the other hand, studies with [11C]PK11195 in patients with brain tumors, AD, stroke and multiple sclerosis, indicated improved *in vivo* pharmacokinetics as a PET radiotracer compared with [11C]Ro5‐4864, however, it too displayed poor signal to noise ratio, low blood–brain barrier permeability and high nonspecific binding 158. Attempts to enhance the pharmacokinetic properties of PK11195 through derivatization as well as labeling with the longer lived PET isotope fluorine‐18 or the single photon emission computer tomography (SPECT) isotope iodine‐123 were unsuccessful.

### Radiolabeled TSPO ligands

A considerable number of diverse chemical entities with high specificity and selectivity for TSPO have been developed in the last 30 years 152. All of these molecules were identified by their ability to compete for binding sites against Ro5‐4864 and PK11195. As expected, the emergence of these diverse classes of TSPO molecules has also prompted the development of radiolabeled TSPO analogues using [11C]carbon, [123I]iodine and [18F]fluorine. Consequently, more than 50 new TSPO radioligands have been reported [for a review see 41, 42] with most being reported in the last decade and a trend toward [18F]TSPO radioligands. A considerable number of these radioligands have been developed to study the various conditions associated with altered TSPO expression using PET and the related imaging modality SPECT. Furthermore, the combination of TSPO radiotracer formulations with high specific activity and low pharmacological doses has had the added benefit of enabling truly noninvasive PET or SPECT *in vivo* imaging, accelerating the progression to *in vivo* human studies.

The currently leading TSPO radiotracers include the N‐benzyl‐N‐(2‐phenoxyaryl)‐acetamide derivative [11C]‐DAA1106 79, 111, 184, the structural analogues [11C] pyridinylacetamides, [18F]PBR06 64, [18F]FEPPA 178, 2‐phenylpyrazolo[1,5‐a]pyrimidineacetamide [11C]DPA‐713 80, 157 and the 2‐phenylimadzo[1,2‐a]pyridines [123I]‐CLINDE 114 and [18F]PBR111 63. These have been developed with enhanced pharmacological and pharmacokinetic properties and reduced nonspecific binding suitable for imaging TSPO receptors *in vivo* using PET or SPECT. Furthermore, many of these radiotracers have demonstrated superior imaging capability in imaging inflammatory processes in both animal models of disease and in humans in comparison with Ro5‐4864 and PK11195. However, despite the considerable improvements exhibited by these TSPO radiotracers, most developed to date are less than ideal, primarily because of a significant component of nonspecific binding stemming from an unfavorable metabolism and nonspecific uptake of the radiolabeled fragments in regions of interest.

Modeling studies have suggested three common hydrophobic interactions with the tryptophan residues (W42, W107 and W161) and a polar hydrogen bond interaction as a requirement for affinity to TSPO 8, 43, 130. However, the same types of interactions are also anticipated for the CBR with the main difference being the spatial positioning of these interactions 8. Despite the limitation of radionuclidic half‐life (t = 20 minutes) and the complexity of radiolabeling, nearly half of the pool of TSPO radiotracers reported are [11C]carbon analogues. In general, convenience and the need for preservation of the overall chemical structure have been arguments in favor of [11C]carbon radiolabeling. The latter has mainly been through the methylation on a hetero atom such as a nitrogen, as in the first PET TSPO radiotracers [11C]Ro5‐4864 and [11C]PK11195 39, or oxygen, as in [11C]DAA1106 183, to provide the structurally identical analogue. The introduction of other isotopes such as [18F]fluorine or [123I]iodine onto any of the large variety of TSPO chemical structures available requires considerable knowledge of the TSPO pharmacophore and extensive structure‐activity studies of the chemical structure under consideration. Even then, the introduction of [123I]iodine or [18F]fluorine onto existing molecules for more convenient radiochemistry, wider tracer distribution or extended SPECT or PET imaging, respectively, may introduce pharmacological, biological and pharmacokinetic properties that are not always predictable. A clear example of this is in the comparison between [11C]zolpidem and the analogous [123I]iodozlopidem. [11C]zolpidem is a CBR selective ligand that is labeled with [11C]carbon via methylation of the corresponding dimethylacetamide 55. Substitution of the phenyl methyl group of zoplidem with a phenyl iodo group gives iodozolpidem. *In vivo* studies of radiolabeled [123I]iodozolpidem, in contrast to [11C]zolpidem, showed that the modification resulted in a ligand with characteristics of a TSPO ligand 89, 115. It was this important discovery together with the limited pharmacological data derived from Alpidem that significantly influenced our research and development on TSPO‐specific molecules based on the imidazopyridine type structure (a selection of TSPO ligands is listed in Table S4 of Supporting Information).

### Radioligands of the imidazopyridine family

**Figure 10:**
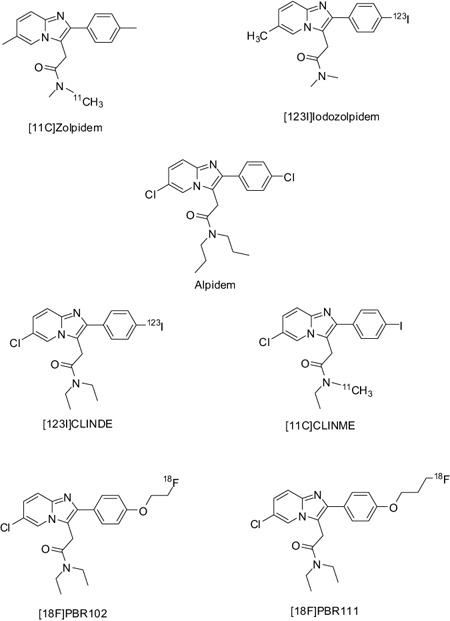
Ligands from the imidazopyridine class.

In the last two decades, our group has developed several TSPO‐selective radioligands that could be used for PET or SPECT imaging; an extensive structure‐activity study of molecular entities spanning more than 10 different chemical families resulted in the synthesis and evaluation of over 250 novel molecules 50, 86, 88, of which over 50 displayed high nanomolar affinity for TSPO with excellent selectivity over the CBR (Figure 10). From these, 22 novel radiotracers bearing carbon‐11 [11C], fluorine‐18 [18F] and iodine‐123 [123I] representing the imidazopyridines 87, 88, [123I]CLINDE 87, [11C]CLINME 169 and [123I]CLINME 116; the structurally related imidazopyridazines 88, 2‐phenylpyrazolo[1,5‐a]pyrimidine acetamides 63 and [123I] N,N‐dialkyl‐2‐phenylindol‐3‐yl‐glyoxylamides 78; as well as the N‐benzyl‐N‐(2‐phenoxyaryl)‐acetamide derivatives, were prepared and extensively evaluated in rodents, with many of these compounds displaying improved biological characteristics, such as a higher target to background signal, faster clearance from nonspecific tissue and improved metabolic stability relative to [11C]PK11195 in *in vivo* animal studies 32, 52, 55, 63, 115. The imidazopyridines were the most successful; five of these radiotracers ([123I]‐CLINDE, [123I]‐CLINME, [11C]‐CLINME, [18F]‐PBR111 and [18F]‐PBR102) have further been evaluated in several rodent animal models of disease 10, 11, 33, 113, 114, 160, 171 confirming that these radiotracers can image and measure activated microglia/macrophage infiltration in the CNS. In addition, we have combined and compared the results of *in vitro* ligand binding, autoradiography, RT‐qPCR (reverse transcription quantitative real‐time polymerase chain reaction) and immunohistochemical staining with *in vivo* small animal SPECT and PET imaging using our novel radiotracers [123I]CLINDE 113, 114 and [18F]PBR111 in models of multiple sclerosis in rodents (experimental autoimmune encephalomyelitis, EAE) 117. In this work, we have demonstrated uptake of radioactivity in the spinal cord and brain tissues from rodents with EAE, which directly correlated to sites of inflammatory lesions and immunoreactivity for the activated microglia/macrophage and astroglia markers confirming the use of *in vitro* and *in vivo* ligand binding to TSPO as a measure of lesion activity in autoimmune CNS demyelinating diseases. Most importantly, TSPO imaging using these radiotracers enabled us to measure and quantify TSPO expression in activated microglia with excellent correlation to *in vitro* immunohistological staining and RT‐qPCR results 117. Four tracers ([123I]‐CLINDE, [18F]PBR102, [18F]‐PBR111 and [18F]PBR170) were progressed to pharmacokinetic studies involving nonhuman primates while the SPECT ligand [123I]CLINDE and the PET ligand [18F]PBR111 were found to display some of the most favorable *in vivo* properties 175. These studies (unpublished data) indicate that unlike the results obtained from rodents, both [18F]PBR102 and [18F]PBR111 display similar pharmacokinetics in primates with some defluorination observed for [18F]PBR111. The expected formation of [18F]fluoroacetate following oxidative metabolism of the O‐ethyl group of PBR102, although present, was not as significant in primates as that observed in rodents. The promising preclinical data for [123I]CLINDE and [18F]PBR111 have allowed these compounds to become candidates for further evaluation in humans with neurodegenerative diseases such as Parkinson's and multiple sclerosis 44, 72, 185.

Radioligands are often metabolized by experimental subjects during imaging. The metabolism of the ligands is thus a parameter that must be considered for imaging quantification. Resulting radiometabolites that also have affinity to the receptor being imaged will distort the analysis, whereas high levels of radiometabolites that enter the region of interest will decrease the potential sensitivity of the radioligand. Strategic positioning of the radiolabel can moderate these troublesome radiometabolites; however, knowing the site of metabolic cleavage on the molecule is not always predictable and is further complicated by species differences 145.

### Metabolite studies

**Figure 11:**
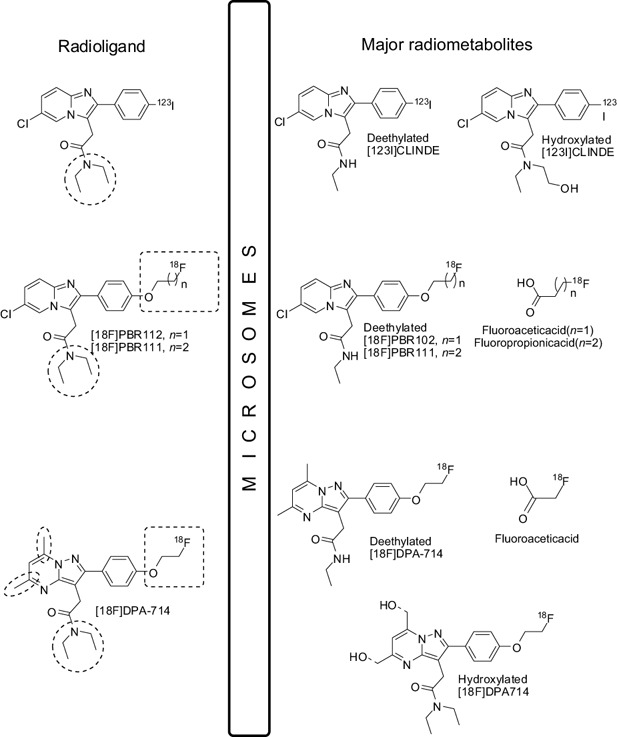
The major metabolites of the [123I]CLINDE, [18F]PBR102, [18F]PBR111 and [18F]DPA714.

*In vitro* metabolic studies of the TSPO ligands CLINDE, PBR111, PBR102 and the bioisosterically similar compound DPA‐714, using rat and human microsomes, have identified that the common main metabolic pathway in this group is via N‐dealkylation, O‐dealkylation and hydroxylation of aliphatics 142, 143, 144. N‐deethylation and hydroxylation derivatizes these ligands into radiometabolites that resemble the parent (unmetabolized ligand) and can compete with the receptor (Figure 11). Our work has revealed that the N‐deethylated [123I]CLINDE derivative has affinity for TSPO (PBR IC50 = 10 nM, CBR IC50 = 104 nM) (unpublished data). This may also have implications for other dialkylated secondary amino TSPO radioligands, where the radiolabel is part of the N‐dealkylated product.

As previously mentioned, many of the TSPO ligands were derived from [11C]methylation of a nitrogen or an oxygen. Dealkylation of these methyl groups results in the formation of [11C]formaldehyde and [11C]carbon dioxide, which for brain studies, does not interfere as these radiometabolites do not cross the blood–brain barrier. In contrast, several of the [18F]fluorinated TSPO radioligands are [18F]‐O‐fluoroalkylated derivatives (PBR102, PBR111, DPA714, FEPPA and FEDAA1106). O‐dealkylation can result in an [18F]fluorinated aldehydes which can subsequently be oxidized to their [18F]fluorinated acids. Further metabolism of these acids can result in [18F]fluoride which can accumulate in bone potentially complicating the imaging analysis and decreasing the ability of the radioligand to identify subtle changes to the receptor. However, the extent of metabolism is dependent on species 142, 145; while it may present a problem in one species, it may not be as significant in another.

**Figure 12:**
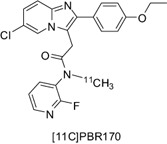
Structure of PBR170.

To further refine our established PET radioligands and considering the metabolic data, our group synthesized a new [11C]imidazopyridine ligand, [11C]PBR170 (PBR IC50 4.5 nM, CBR IC50 > 10 000 nM) 31, 56 (Figure 12). Similar to [11C]CLINME, the [11C]carbon resides on the acetamide as a [11C]methyl group. The other group on the acetamide is a fluoro pyridine which serves to prevent the N‐deethylation observed with previous series of TSPO ligands. It also allows potential introduction of [18F]fluorine into the ligand via the fluoro pyridine as an option for longer imaging times and also offers a comparison between the different radiometabolites of the same tracer. In general, an aromatic fluoride is less susceptible to defluorination. Although O‐deethylation may occur, the resulting phenolic radiometabolite is expected to be more water soluble and unlikely to enter the brain, while at the same time having a faster clearance rate.

Preliminary results show that [11C]PBR170 shows saturable, displaceable and specific binding in TSPO‐rich tissues. The kinetics and low nonspecific cerebral binding make this radiotracer suitable for imaging TSPO‐associated brain abnormalities 56.

Another complexity in assessing radioligand potential in humans is due to a genetic polymorphism of TSPO 93, 132; there are three types of binders within the population: high‐, low‐ and mixed‐affinity binders 93, 131. The radioligands PBR28, PBR06 and DAA1106 differentiate between the high‐ and low‐affinity binders by approximately fifty‐, seventeen‐ and four‐fold, respectively, despite all belonging to the same class of TSPO radioligands 131. In contrast, PK11195 binds at comparable levels across the population. PBR111 and DPA713 were also evaluated and showed an approximate fourfold difference. These findings have not deterred further clinical evaluation of [18F]PBR111 in multiple sclerosis patients 44, 146 as it still offers other advantages over PK11195.

## Future Directions for TSPO Imaging Ligands

Imaging the subtle changes in TSPO expression by PET or SPECT requires the design of radioligands with exquisite sensitivity and specificity, free of deleterious signal contributed by spurious metabolites. Accordingly, attempts to image diseases using radiotracers that compromise this fine signal would be futile. With so many applications and a plethora of novel TSPO radiotracers available, it is an exciting time to extract the clinical value that TSPO has to offer. Of the radiotracers that have already progressed to human studies for neuroinflammation and neurodegenerative diseases 42, there is still no single tracer that offers a clear advantage over the rest for widespread use. This highlights the need to further explore these TSPO radiotracers in the clinic while attempting to further elucidate the nature of TSPO itself. With so many questions to be answered, a better understanding of the relationship between these molecules and the biological function of TSPO is required. It also appears that we may have exhausted the potential for ligand–receptor interactions to further characterize this enigmatic protein and focus now should be on its biological characterization by other means.

## Conclusion

A substantial part of the research relating to TSPO is focused on its diagnostic utility in neuroimaging. It is, therefore, possible to interpret the development of scientific knowledge around “TSPO and neuroimaging” so far as the appropriation of concepts from different fields. Starting with the rediscovery of microglia in the 1960s, the wider availability of immunohistochemical staining techniques for microglia in the early 1990s led to the gradual recognition that the brain was not an “immune‐privileged” organ and catalyzed the evolution of the “neuroinflammation” concept. Since then, the “neuroinflammation” concept has found steadily broadening applications, including to illnesses that traditionally have been accompanied by disagreement in regard to construct validity and nosology, such as schizophrenia 14, 36. Somewhat similarly, the notion of “translocation” in the context of TSPO was able to gather the many strands of evidence into a more unified theory, where previously the role of TSPO could best be described as “enigmatic” 67.

In the discussion around “neuroinflammation” and the “translocator protein,” one witnesses the role and impact of lexical changes in new scientific developments 148, which provides “pragmatic ambiguity” 70 in the face of incomplete mechanistic understanding. The problem of specific annotations of gene functions is well recognized 66. More importantly, however, these controversies point to a fundamental challenge in biology, namely deciding the appropriate level at which a description allows conversion of “data into knowledge and knowledge into understanding” 35. Research into TSPO is arguably confronted with the classical inverse problem; that is, how to arrive at a model of TSPO function from observations at a higher level of complexity, for example behavior. In this context, the theory that TSPO is a “translocator” promised a welcome reduction in complexity. Yet, the one important forward prediction based on this theory, namely that a full *Tspo* knockout mouse should either be embryonic lethal, as in fact had been reported 134, or substantially impaired because of impaired steroid synthesis, has not held true. On the other hand, from a “systems biological” perspective, the observation that *Tspo* is an ancient, nonessential gene could be interpreted as support that its function is in fact an emergent property that necessarily requires the context of other pathways and protein–protein interactions.

Drug discovery marked the beginning of research into TSPO and its role in physiology. With the availability of well‐characterized *Tspo* knockout animals with normal physiology across their lifespan 18, 19, a new opportunity is at hand to create better investigational tools in the form of highly selective compounds for TSPO. Because of the lipophilic nature of many TSPO ligands, they can integrate into the lipid membranes of cells and organelles with ease and, we speculate, cause functional responses 75, 139. We, therefore, anticipate innovative studies into the biophysical mechanisms by which selective ligands for the PBR/TSPO may exert either modified or additional actions in the presence or absence of TSPO, using the unique opportunity to compare normally functioning cells and organelles from wild‐type and knockout animals. Such studies have the potential to make a broader contribution to the understanding of drug activity, where ligand–membrane interactions may explain functional effects that receptor–ligand interactions alone cannot.

## Supporting information


**Figure S1.** (**A**) Publication of articles within search terms; refined by name used for TSPO. Evidence indicates Ro5‐4864 has been replaced by newer ligands targeting TSPO; however, PK11195 is still widely used as the prototypical TSPO ligand. In spite of the change in nomenclature, TSPO is still very commonly referred to in terms of its pharmacological properties (ie, the ability to bind benzodiazepines in the periphery). (**B**) Citation of articles within search terms each year; sorted by name used for TSPO. Articles using the term “peripheral benzodiazepine,” “PK11195/PK 11195” and “peripheral‐type benzodiazepine” receive considerably more citations than articles exclusively using the other search terms.
**Table S1.** Clinical trials relating to neuroinflammation using TSPO ligands (from clinicaltrials.gov).
**Table S2.** Terms for literature search.
**Table S3.** TSPO‐associated proteins.
**Table S4.** TSPO ligands.
