# Supplementary material for: The 18 kDa Translocator Protein, Microglia and Neuroinflammation
Source: Brain Pathol. 2014 Oct 26;24(6):631–53. doi: 10.1111/bpa.12196 (PMC8029074; doi:10.1111/bpa.12196)
Supplement: Supplementary file 1 — Figure S1. (A) Publication of articles within search terms; refined by name used for TSPO. Evidence indicates Ro5‐4864 has been replaced by newer ligands targeting TSPO; however, PK11195 is still widely used as the prototypical TSPO ligand. In spite of the change in nomenclature, TSPO is still very commonly referred to in terms of its pharmacological properties (ie, the ability to bind benzodiazepines in the periphery). (B) Citation of articles within search terms each year; sorted by name used for TSPO. Articles using the term “peripheral benzodiazepine,” “PK11195/PK 11195” and “peripheral‐type benzodiazepine” receive considerably more citations than articles exclusively using the other search terms. Table S1. Clinical trials relating to neuroinflammation using TSPO ligands (from clinicaltrials.gov). Table S2. Terms for literature search. Table S3. TSPO‐associated proteins. Table S4. TSPO ligands. [file BPA-24-631-s001.docx]

**Supplementary Material:**

**Figure 1**

**A**


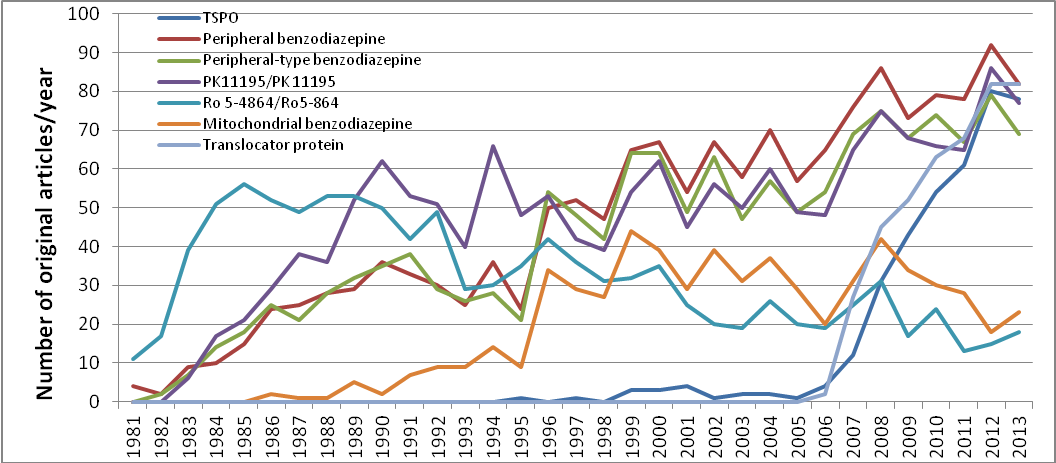


**B**


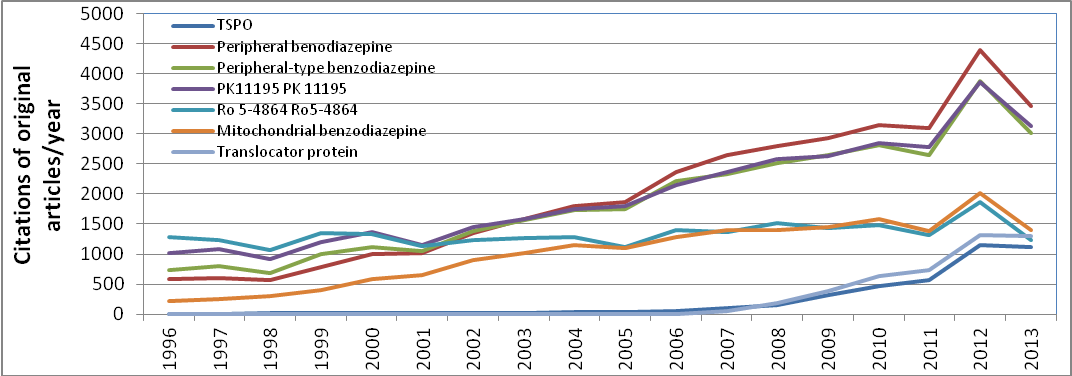


(A) Publication of articles within search terms; refined by name used for TSPO. Evidence indicates Ro5-4864 has been replaced by newer ligands targeting TSPO; however PK-11195 is still widely used as the prototypical TSPO ligand. In spite of the change in nomenclature, TSPO is still very commonly referred to in terms of its pharmacological properties (that is the ability to bind benzodiazepines in the periphery).

(B) Citation of articles within search terms each year; sorted by name used for TSPO. Articles using the term ‘peripheral benzodiazepine’, ‘PK11195/PK 11195’, and ‘peripheral-type benzodiazepine’ receive considerably more citations than articles exclusively using the other search terms.

**Table 1. Clinical trials relating to neuroinflammation using TSPO ligands (from clinicaltrials.gov)**

| **Title** | **Conditions** | **TSPO PET ligand** | **Clinicaltrials.gov identifier** |
| --- | --- | --- | --- |
| Evaluation of Neuroinflammation in Children With Infantile Spasms | Infantile Spasms | [11C]PK11195 | NCT02092883 |
| PET Imaging of the Translocator Proteine Ligands (TSPO) With [18 F] DPA-714 Biomarker of NeuroInflammation in Cognitive Decline (NIDECO) | Memory Complaint, Mild Cognitive Impairment, Alzheimer Disease | [18F]DPA-714 | NCT02062099 |
| Study to Assess Neuroinflammation and Neurocognitive Function in Patients With Acute Hepatitis C and Chronic HIV Co-Infection - A Positron Emission Tomography (PET) Study | Acute Hepatitis C/HIV, HIV Infections | [11C]PK11195 | /NCT00959166 |
| Postoperative Neuroinflammation and Cognitive Dysfunction After Abdominal Surgery | Anesthesia,Surgery, Neurogenic Inflammation, Cognitive Disorders | [11C]PBR28 | NCT01881646 |
| Psychosis-Associated Neuroinflammation in Schizophrenia | Schizophrenia, Psychosis | [18F]PBR111 | NCT02009826 |
| Evaluation of the Neuroinflammation Pattern of BAY85-8102 F-18, DPA-714 in Probable Alzheimers Disease Patients Versus Healthy Volunteers and Radiation Dosimetry of F 18, DPA-714 in Healthy Volunteers | Diagnostic Imaging | [18F]DPA-714 | NCT01009359 |
| Brain P-gp and Inflammation in People With Epilepsy | Epilepsies, Partial | PBR28 | NCT01663545 |
| Inflammation and Electroconvulsive Therapy | Major Depressive Disorder | [18F]FEPPA | NCT02095639 |
| Molecular Imaging Modality by Positron Emission Tomography Using 18F-X : Study of Microglial Activation in Amyotrophic Lateral Sclerosis | Amyotrophic Lateral Sclerosis, Bulbar Disease, Spinal Disease | [18F]DPA-714 | NCT00563537 |
| PET Imaging of Peripheral Benzodiazepine Receptors in Patients With Neurocysticercosis Using [C-11]PBR28 | Neurocysticercosis, Healthy | [11C]PBR28 | NCT00526916 |
| Brain Inflammation in Major Depressive Disorder Background | Major Depression | [11C]PBR28 | NCT01851356 |
| Evaluation of ZK 6032924 in Probable Alzheimer's Disease Patients Versus Healthy Volunteers and the Radiation Dosimetry of ZK 6032924 in Healthy Volunteers | Positron-Emission Tomography, Alzheimer's Disease | [18F]FEDAA1106 | NCT01035164 |
| PET Imaging of Peripheral Benzodiazepine Receptors | Healthy | [11C](R)-PK 11195, [11C]PBR28 | NCT00696371 |
| PET Imaging of Peripheral Benzodiazepine Receptors in Patients With Neurocysticercosis Using [F-18]FBR | Neurocysticercosis, Healthy | [18F]FBR | NCT00527579 |
| To Rescue Cognition With Valaciclovir | Schizophrenia, Psychosis | [11C]PK11195 | NCT01364792 |
| Evaluation of the Positron Emission Tomography (PET) Tracer ZK 6032924 in Patients With Multiple Sclerosis Compared to Healthy Volunteers | Positron-Emission Tomography, Multiple Sclerosis | [18F]FEDAA1106 | NCT01031199 |
| Efficacy, Safety and Tolerability of XBD173 in Patients With Generalized Anxiety Disorder* | Anxiety Disorders | XBD173 | NCT00108836 |

********(TSPO is not listed explicitly as the drug target, but has been indicated previously (*[*26*](#_ENREF_26)*))*

**Table 2. Terms for literature search**

| **Search terms (TITLE-ABS-KEY; “OR”)**  **Document type (Articles and Reviews)** | Results (as of 23.7.2014) |
| --- | --- |
| TSPO | 2763 |
| “peripheral benzodiazepine” |  |
| “peripheral-type benzodiazepine” |  |
| PK11195 |  |
| “PK 11195” |  |
| Ro5-4864 |  |
| “Ro 5-4864” |  |
| “mitochondrial benzodiazepine” |  |

SCOPUS search terms for TSPO literature, optimized for lowest proportion of non-relevant articles. It was not possible to include “translocator protein” as an isolated search term, as a high proportion of results were relating to other ‘translocator proteins.’

**Table 3. TSPO associated proteins.**

| **Symbol** | **Name** | **Association with TSPO** | **Reference** |
| --- | --- | --- | --- |
| TSPO2 | translocator protein 2 | Paralog of TSPO that arose before avians and mammals diverged | ([10](#_ENREF_10)) |
| VDAC | voltage-dependant anion channel | Part of a multimeric complex with TSPO | ([21](#_ENREF_21)) |
| ANT | adenine nucleotide translocator | Part of a multimeric complex with TSPO | ([21](#_ENREF_21)) |
| BZRAP1 (PRAX-1) | benzodiazapine receptor (peripheral) associated protein 1 | Interacts with TSPO | ([13](#_ENREF_13)) |
| StAR | steroidogenic acute regulatory protein | Interacts with TSPO to facilitate the transport of cholesterol | ([31](#_ENREF_31)) |
| ACBD3 (PAP7) | acyl-CoA binding domain containing 3 | Binds to TSPO and is implicated in steroid biosynthesis | ([20](#_ENREF_20)) |
| PRKAR1A **(**PKA-R1alpha) | protein kinase, cAMP-dependent, regulatory, type I, alpha | Binds to ACBD3 bringing PRKAR1A to mitochondria | ([20](#_ENREF_20)) |
| PKA | Protein kinase A | Phosphorylation of TSPO | ([32](#_ENREF_32)) |
| pk 10 | Unknown 10 kDa protein | In a complex with TSPO and can bind to TSPO ligands | ([4](#_ENREF_4)) |
| DBI | diazepam binding inhibitor (GABA receptor modulator, acyl-CoA binding protein) | Binds to TSPO and acts as an endogenous ligand | ([14](#_ENREF_14)) |
| Porphyrins | - | Endogenous ligands for the TSPO | ([30](#_ENREF_30)) |
| OPA1 | optic atrophy 1 (autosomal dominant) | Present in a 800 kDa complex that included TSPO | ([25](#_ENREF_25)) |
| LONP1 | lon peptidase 1, mitochondrial | Present in a 800 kDa complex that included TSPO | ([25](#_ENREF_25)) |
| C8orf17 | chromosome 8 open reading frame 17 | Interacts with TSPO in yeast 2 hybrid screen | ([28](#_ENREF_28)) |
| ATAD3A | ATPase family, AAA domain containing 3A | Present in a 800 kDa complex that included TSPO | ([25](#_ENREF_25)) |
| CYP11A1 | cytochrome P450, family 11, subfamily A, polypeptide 1 | Present in a 800 kDa complex that included TSPO | ([25](#_ENREF_25)) |
| ACAT1 | acetyl-CoA acetyltransferase 1 | Present in a 800 kDa complex that included TSPO | ([25](#_ENREF_25)) |
| IDH2 | isocitrate dehydrogenase 2 (NADP+), mitochondrial | Present in a 800 kDa complex that included TSPO | ([25](#_ENREF_25)) |
| MDH2 | malate dehydrogenase 2, NAD (mitochondrial) | Present in a 800 kDa complex that included TSPO | ([25](#_ENREF_25)) |
| FDXR | ferredoxin reductase | Present in a 66 kDa complex that included TSPO | ([25](#_ENREF_25)) |
| PLA2 | Phospholipase A2 | Reduced the affinity of a ligand to TSPO | ([16](#_ENREF_16)) |

**Table 4. TSPO ligands**

| Class | Example Name | IUPAC | How identified | Reference |
| --- | --- | --- | --- | --- |
| Benzodiazepine | Diazepam | 7-chloro-1-methyl-5-phenyl-3H-1,4-benzodiazepin-2-one | Binding of hot ligand in peripheral tissues of rat | ([6](#_ENREF_6)) |
| Benzodiazepine | Ro 5-4864 | 7-chloro-5-(4-chlorophenyl)-1-methyl-3H-1,4-benzodiazepin-2-one | Competition studies against diazepam | ([6](#_ENREF_6)) |
| Isoquinoline carboxamide | PK 11195 | 1-(2-chlorophenyl)-N-methyl-N-sec-butyl-isoquinoline-3-carboxamide | Competetion studies against [3H]Ro 5-4864 | ([19](#_ENREF_19)) |
| Imidazopyridine acetamide | Alpidem | 2-[6-chloro-2-(4 chlorophenyl)imidazo [1,2-a]pyridin-3-yl]-N,N-diethyl-acetamide | Competition against [3H]diazepam and [3H]Ro 5-4864 | ([18](#_ENREF_18)) |
| Arylindol acetamide | FGIN-1-27 | 2-[2-(4-fluorophenyl)-1H-indol-3-yl]-N,N-dihexyl-acetamide | Competition studies against [3H]diazepam and [3H]PK 11195 | ([24](#_ENREF_24)) |
| Benzothiazepine | THIA-66 | [6-(4-methoxyphenyl)pyrrolo[2,1-d][1,5] benzothiazepin-7-yl] acetate | Competition studies against [3H]PK 11195 | ([12](#_ENREF_12)) |
| Benzoxazepine | OXA-17f | (6-phenylpyrrolo[2,1-d][1,5]benzoxapin-7-yl) N,N-diethylcarbamate | Competition studies against [3H]Ro 5-4864 and [3H]PK 11195 | ([9](#_ENREF_9)) |
| Phenoxyphenyl acetamide | DAA1106 | N-[(2,5-dimethoxyphenyl0methyl]-N-(5-fluoro-2-phenoxy-phenyl) acetamide | Competition studies against [3H]Ro 5-4864 and [3H]PK 11195 | ([22](#_ENREF_22)) |
| Pyrazolopyrimidine acetamide | DPA-713 | N,N-diethyl-2-[2-(4-methoxyphenyl)-5,7-dimethyl-pyrazolo [1,5-a]pyrimidin-3-yl] acetamide | Competition studies against [3H]Ro 5-4864 and [3H]PK 11195 | ([27](#_ENREF_27)) |
| Pyridazinoindole acetamide | SSR180575 | 2-(7-chloro-5-methyl-4-oxo-3-phenyl-pyridazino[4,5-b]indol-1-yl)-N,N-dimethyl-acetamide | Competition studies against [3H]Ro 5-4864 and [3H]PK 11195 | ([11](#_ENREF_11)) |
| Phenylpurine acetamide | Emapunil (AC-5216/XBD171) | N-benzyl-N-ethyl-2-(7-methyl-8-oxo-2-phenyl-purin-9-yl) acetamide | Competition studies against [3H]PK 11195 | ([17](#_ENREF_17)) |
| Phenylindolylglyoxylamide | MPIGA | N,N-di-n-propyl-2-(4-methylphenyl)indol-3-ylglyoxylamide | Competition studies against [3H]PK 11195 | ([23](#_ENREF_23)) |
| Phenoxyphenyl acetamide | PBR01 | methyl 2-[(N-acetyl-2-phenoxy-anilino)methyl] benzoate | Displacement of PK 11195 and DAA1106 in PET imaging studies. | ([7](#_ENREF_7)) |
| Alkaloid | Vinpocetine | (3α, 16α)-Eburnamenine-14-carboxylic acid ethyl ester | PET competition against PK 11195 | ([15](#_ENREF_15)) |
| Benzoxazine | Etifoxine | 6-chloro-2-(ethyl-amine)-4-methyl-4-phenyl-4H-3,l-benzoxazine | Competition studies against [3H]PK 11195 | ([29](#_ENREF_29)) |
| Imidazopyridine acetamide | CLINME | 2-[6-chloro-2-(4-iodophenyl)imidazo[1,2-a]pyridin-3-yl]-N-ethyl-N-methyl-acetamide | Displacement of PK 11195 in PET imaging studies. | ([5](#_ENREF_5)) |

**References (Supplementary Material)**

1. Banati RB (2002) Visualising microglial activation in vivo. Glia.40(2):206-17.

2. Banati RB (2003) Neuropathological imaging: in vivo detection of glial activation as a measure of disease and adaptive change in the brain. British medical bulletin.65:121-31.

3. Banati RB, Myers R, Kreutzberg GW (1997) PK ('peripheral benzodiazepine')--binding sites in the CNS indicate early and discrete brain lesions: microautoradiographic detection of [3H]PK11195 binding to activated microglia. Journal of neurocytology.26(2):77-82.

4. Blahos J, 2nd, Whalin ME, Krueger KE (1995) Identification and purification of a 10-kilodalton protein associated with mitochondrial benzodiazepine receptors. J Biol Chem.270(35):20285-91.

5. Boutin H, Chauveau F, Thominiaux C, Kuhnast B, Gregoire MC, Jan S, Trebossen R, Dolle F, Tavitian B, Mattner F, Katsifis A (2007) In vivo imaging of brain lesions with [(11)C]CLINME, a new PET radioligand of peripheral benzodiazepine receptors. Glia.55(14):1459-68.

6. Braestrup C, Squires RF (1977) Specific benzodiazepine receptors in rat brain characterized by high-affinity (3H)diazepam binding. Proceedings of the National Academy of Sciences of the United States of America.74(9):3805-9.

7. Briard E, Zoghbi SS, Imaizumi M, Gourley JP, Shetty HU, Hong J, Cropley V, Fujita M, Innis RB, Pike VW (2007) Synthesis and evaluation in monkey of two sensitive 11C-labeled aryloxyanilide ligands for imaging brain peripheral benzodiazepine receptors in vivo. Journal of medicinal chemistry.51(1):17-30.

8. Cagnin A, Brooks DJ, Kennedy AM, Gunn RN, Myers R, Turkheimer FE, Jones T, Banati RB (2001) In-vivo measurement of activated microglia in dementia. Lancet.358(9280):461-7.

9. Campiani G, Nacci V, Fiorini I, De Filippis MP, Garofalo A, Ciani SM, Greco G, Novellino E, Williams DC, Zisterer DM, Woods MJ, Mihai C, Manzoni C, Mennini T (1996) Synthesis, biological activity, and SARs of pyrrolobenzoxazepine derivatives, a new class of specific "peripheral-type" benzodiazepine receptor ligands. Journal of medicinal chemistry.39(18):3435-50.

10. Fan J, Rone MB, Papadopoulos V (2009) Translocator protein 2 is involved in cholesterol redistribution during erythropoiesis. J Biol Chem.284(44):30484-97.

11. Ferzaz B, Brault E, Bourliaud G, Robert JP, Poughon G, Claustre Y, Marguet F, Liere P, Schumacher M, Nowicki JP, Fournier J, Marabout B, Sevrin M, George P, Soubrie P, Benavides J, Scatton B (2002) SSR180575 (7-chloro-N,N,5-trimethyl-4-oxo-3-phenyl-3,5-dihydro-4H-pyridazino[4,5-b]indole-1 -acetamide), a peripheral benzodiazepine receptor ligand, promotes neuronal survival and repair. The Journal of pharmacology and experimental therapeutics.301(3):1067-78.

12. Fiorini I, Nacci V, Ciani SM, Garofalo A, Campiani G, Savini L, Novellino E, Greco G, Bernasconi P, Mennini T (1994) Novel ligands specific for mitochondrial benzodiazepine receptors: 6-arylpyrrolo[2,1-d][1,5]benzothiazepine derivatives. Synthesis, structure-activity relationships, and molecular modeling studies. Journal of medicinal chemistry.37(10):1427-38.

13. Galiegue S, Jbilo O, Combes T, Bribes E, Carayon P, Le Fur G, Casellas P (1999) Cloning and characterization of PRAX-1. A new protein that specifically interacts with the peripheral benzodiazepine receptor. J Biol Chem.274(5):2938-52.

14. Garnier M, Boujrad N, Ogwuegbu SO, Hudson JR, Jr., Papadopoulos V (1994) The polypeptide diazepam-binding inhibitor and a higher affinity mitochondrial peripheral-type benzodiazepine receptor sustain constitutive steroidogenesis in the R2C Leydig tumor cell line. J Biol Chem.269(35):22105-12.

15. Gulyás B, Halldin C, Vas Á, Banati RB, Shchukin E, Finnema S, Tarkainen J, Tihanyi K, Szilágyi G, Farde L (2005) [11C] Vinpocetine: a prospective peripheral benzodiazepine receptor ligand for primate PET studies. Journal of the neurological sciences.229:219-23.

16. Havoundjian H, Cohen RM, Paul SM, Skolnick P (1986) Differential sensitivity of "central" and "peripheral" type benzodiazepine receptors to phospholipase A2. Journal of neurochemistry.46(3):804-11.

17. Kita A, Kohayakawa H, Kinoshita T, Ochi Y, Nakamichi K, Kurumiya S, Furukawa K, Oka M (2004) Antianxiety and antidepressant-like effects of AC-5216, a novel mitochondrial benzodiazepine receptor ligand. Br J Pharmacol.142(7):1059-72.

18. Langer SZ, Arbilla S (1988) Imidazopyridines as a tool for the characterization of benzodiazepine receptors: A proposal for a pharmacological classification as omega receptor subtypes. Pharmacology Biochemistry and Behavior.29(4):763-6.

19. Le Fur G, Perrier ML, Vaucher N, Imbault F, Flamier A, Benavides J, Uzan A, Renault C, Dubroeucq MC, Gueremy C (1983) Peripheral benzodiazepine binding sites: effect of PK 11195, 1-(2-chlorophenyl)-N-methyl-N-(1-methylpropyl)-3-isoquinolinecarboxamide. I. In vitro studies. Life Sci.32(16):1839-47.

20. Li H, Degenhardt B, Tobin D, Yao ZX, Tasken K, Papadopoulos V (2001) Identification, localization, and function in steroidogenesis of PAP7: a peripheral-type benzodiazepine receptor- and PKA (RIalpha)-associated protein. Mol Endocrinol.15(12):2211-28.

21. McEnery MW, Snowman AM, Trifiletti RR, Snyder SH (1992) Isolation of the mitochondrial benzodiazepine receptor: association with the voltage-dependent anion channel and the adenine nucleotide carrier. Proc Natl Acad Sci U S A.89(8):3170-4.

22. Okuyama S, Chaki S, Yoshikawa R, Ogawa S, Suzuki Y, Okubo T, Nakazato A, Nagamine M, Tomisawa K (1999) Neuropharmacological profile of peripheral benzodiazepine receptor agonists, DAA1097 and DAA1106. Life sciences.64(16):1455-64.

23. Primofiore G, Da Settimo F, Taliani S, Simorini F, Patrizi MP, Novellino E, Greco G, Abignente E, Costa B, Chelli B, Martini C (2004) N,N-dialkyl-2-phenylindol-3-ylglyoxylamides. A new class of potent and selective ligands at the peripheral benzodiazepine receptor. Journal of medicinal chemistry.47(7):1852-5.

24. Romeo E, Auta J, Kozikowski AP, Ma D, Papadopoulos V, Puia G, Costa E, Guidotti A (1992) 2-Aryl-3-indoleacetamides (FGIN-1): a new class of potent and specific ligands for the mitochondrial DBI receptor (MDR). Journal of Pharmacology and Experimental Therapeutics.262(3):971-8.

25. Rone MB, Midzak AS, Issop L, Rammouz G, Jagannathan S, Fan J, Ye X, Blonder J, Veenstra T, Papadopoulos V (2012) Identification of a dynamic mitochondrial protein complex driving cholesterol import, trafficking, and metabolism to steroid hormones. Mol Endocrinol.26(11):1868-82.

26. Rupprecht R, Rammes G, Eser D, Baghai TC, Schule C, Nothdurfter C, Troxler T, Gentsch C, Kalkman HO, Chaperon F, Uzunov V, McAllister KH, Bertaina-Anglade V, La Rochelle CD, Tuerck D, Floesser A, Kiese B, Schumacher M, Landgraf R, Holsboer F, Kucher K (2009) Translocator protein (18 kD) as target for anxiolytics without benzodiazepine-like side effects. Science (New York, NY).325(5939):490-3.

27. Selleri S, Bruni F, Costagli C, Costanzo A, Guerrini G, Ciciani G, Costa B, Martini C (2001) 2-Arylpyrazolo[1,5-a]pyrimidin-3-yl acetamides. New potent and selective peripheral benzodiazepine receptor ligands. Bioorganic & medicinal chemistry.9(10):2661-71.

28. Tan JM, Chow VT (2007) Cellular expression, localization and interactions of the product of the human MOST-1 gene associated with breast and prostate cancers. Int J Oncol.30(1):81-9.

29. Verleye M, Akwa Y, Liere P, Ladurelle N, Pianos A, Eychenne B, Schumacher M, Gillardin J-M (2005) The anxiolytic etifoxine activates the peripheral benzodiazepine receptor and increases the neurosteroid levels in rat brain. Pharmacology Biochemistry and Behavior.82(4):712-20.

30. Verma A, Nye JS, Snyder SH (1987) Porphyrins are endogenous ligands for the mitochondrial (peripheral-type) benzodiazepine receptor. Proc Natl Acad Sci U S A.84(8):2256-60.

31. West LA, Horvat RD, Roess DA, Barisas BG, Juengel JL, Niswender GD (2001) Steroidogenic acute regulatory protein and peripheral-type benzodiazepine receptor associate at the mitochondrial membrane. Endocrinology.142(1):502-5.

32. Whalin ME, Boujrad N, Papadopoulos V, Krueger KE (1994) Studies on the phosphorylation of the 18 kDa mitochondrial benzodiazepine receptor protein. J Recept Res.14(3-4):217-28.
